# Convergent and divergent genetic changes in the genome of Chinese and European pigs

**DOI:** 10.1038/s41598-017-09061-z

**Published:** 2017-08-17

**Authors:** Jing Wang, Huiying Zou, Lei Chen, Xi Long, Jing Lan, Wenjie Liu, Li Ma, Chao Wang, Xinyu Xu, Liming Ren, Zhenglong Gu, Ning Li, Xiaoxiang Hu, Yaofeng Zhao, Yiqiang Zhao

**Affiliations:** 1 Beijing Advanced Innovation Center for Food Nutrition and Human Health, College of Biological Sciences, China Agricultural University, Beijing, P. R. China; 2 State Key Laboratory of Agrobiotechnology, China Agricultural University, Beijing, P. R. China; 3 Chongqing Academy of Animal Science, Chongqing, P. R. China; 4 Department of Computer and Technology, Tsinghua University, Beijing, P. R. China; 5 School of Life sciences, Tsinghua University, Beijing, P. R. China; 6 Division of Nutritional Sciences, Cornell University, Ithaca, USA; 7 Embryo Biotechnology and Reproduction Laboratory, Institute of Animal Science, Chinese Academy of Agricultural Sciences, Beijing, P. R. China

## Abstract

Since 10,000 BC, continuous human selection has led to intense genetic and phenotypic changes in pig (***Sus scrofa***) domestication. Through whole genome analysis of 257 individuals, we demonstrated artificial unidirectional and bidirectional selection as the primary force to shape the convergent and divergent changes between Chinese domestic pigs (CHD) and European domestic pigs (EUD). We identified 31 genes in unidirectional selection regions that might be related to fundamental domestication requirements in pigs. And these genes belong predominantly to categories related to the nervous system, muscle development, and especially to metabolic diseases. In addition, 35 genes, representing different breeding preference, were found under bidirectional selection for the distinct leanness and reproduction traits between CHD and EUD. The convergent genetic changes, contributing physical and morphological adaption, represent the common concerns on pig domestication. And the divergent genetic changes reflect distinct breeding goals between Chinese and European pigs. Using *ITPR3, AHR* and *NMU* as examples, we explored and validated how the genetic variations contribute to the phenotype changes.

## Introduction

Pigs are among the first animals in the world to be domesticated. A growing body of evidence has identified at least two centers of early pig domestication^1, 2^. China, as the main domestication center in East Asia, has multiple native breeds that have been domesticated beginning 8,000 years ago^3^. European pigs were originally domesticated in the Near East (Anotolia) and were dispersed to Europe by human-mediated migration. The introduced pigs had recurrent hybridization with European local wild boars, resulting in the genetic background of the modern European breeds^4–6^.

Domestication is thought to constitute a continuous process involving a series of phenotypic and genetic changes designed to meet human need^1, 7, 8^. Previous genome scans for positive selection have identified sizeable genes that affect coat color, reproduction, body size, growth and metabolism^7, 9–13^. For example, *NR6A1*, *LCORL*, and *PLAG1* exhibit strong signals of selection for increasing body length, and increase meat production in European domesticated pigs^7^. *ESR1*, as a major quantitative trait locus for litter size, experiences positive selection in Asian domestic pigs^14^. *IGF2*, a gene involved in muscle growth and fat deposition, is extensively selected for the purposes of breeding to increase muscle mass^15^. Series of loci involving in behavioral traits were also under strong selection during pig domestication^16^.

Few studies have focused on comparisons between domesticated breeds and their wild counterparts from one origin^17, 18^. Both Chinese domestic pigs (CHD) and European commercial domestic pigs (EUD) might have been selected for these traits thereby showing increased growth and prolificacy compared to their wild ancestors. Meanwhile, both CHD and EUD are apparently less aggressive and more adaptive to domestication condition^19, 20^. On the other hand, human preferences in breeding strategies could result in distinct traits in different domestic breeds^8^. CHD are generally of higher amount of fat, stronger resistance to diseases, and better reproduction traits, while EUD are renowned for rapid growth and superior leanness.

Comparisons of the independent domestication process of pig in China and Europe provides an opportunity to identify the convergent and divergent genetic changes during domestication and elucidate the genetic variations underlying the altered traits.

In this work, we analyzed a worldwide sample of domestic and wild pigs, utilizing whole genome resequencing data of 257 individuals, to investigate and compare the human selection pattern in China and Europe. Interestingly, a lot of genes discovered in our study show association with human metabolic diseases, indicating that pigs could be used as a good model system for medical research.

## Results

### Sample collection

In this study, we included 257 individuals worldwide, of which whole-genome resequencing data are available, and 8 of them were sequenced in this study. All individuals are divided into five groups: a EUD group, including 97 individuals from four typical commercial domestic breeds of Duroc, Landrace, Yorkshire(Large White) and Pietrain; a EUW group, including 34 individuals of European wild boar; a CHW group, comprising 31 individuals, including wild boars in South and North China, as well as wild boars in Korea, which are genetically and phenotypically similar to the North China wild boars; and a CHD group, comprising 81 individuals representing 14 geographically diverse breeds across China. Finally, *Sus verrucosus*, *Sus cebifrons, Sus celebensis*, *Phacochoerus africanus*, *Sus barbatus*, and Samilda wild boar serve as an outgroup (Supplementary Table S1).

SNP calling was performed for each group, using GATK best practice pipeline based on bam files created by the aligner (Supplementary Table S1). We finally obtained 94 million SNPs in total, and SNPs varied from 14 million to 38 million for each group (Supplementary Table S2).

**Figure 1:**
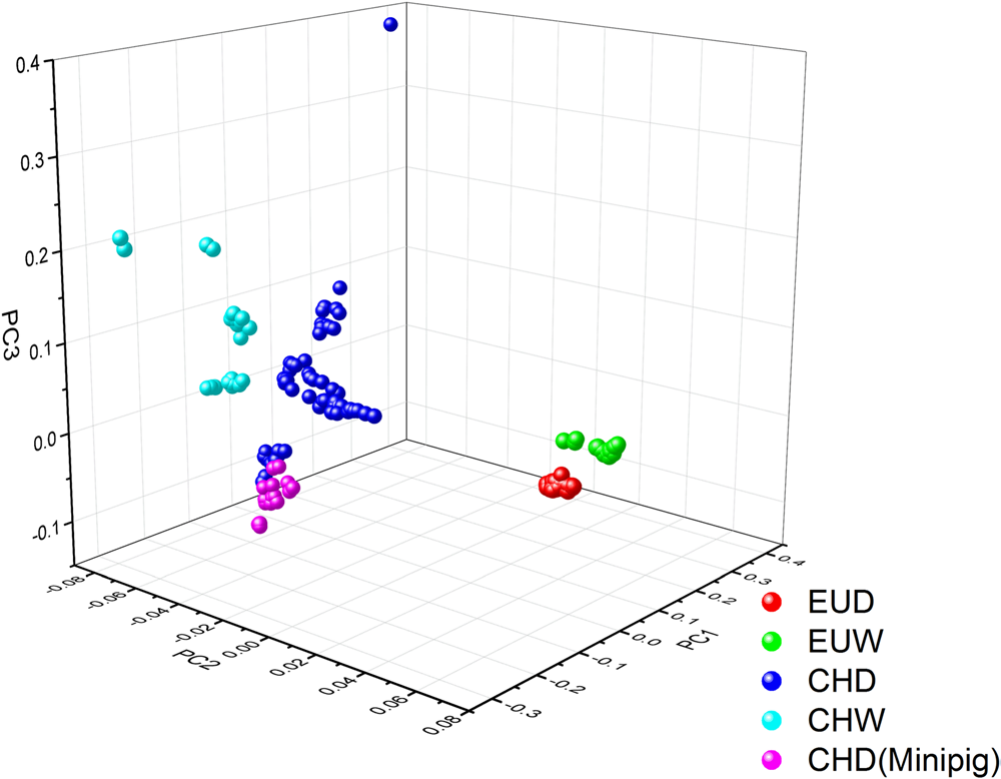
Results of PCA using whole-genome SNP data. Three-way PCA plot of 242 individuals (a Ukraine boar and the Outgroup were excluded in the graph). PC1, principal component 1; PC2, principal component 2; PC3, principal component 3.

Although Chinese wild and domestic pigs exhibited higher genetic variability, principal components analysis (PCA) and IBS (identity-by-state) analysis based on all available SNPs showed that individuals from four defined groups were well separated (Figs 1, Supplementary Fig. 1). 45.6% of the total variance was explained by the first three PCs (PC1, 26%; PC2, 13%; PC3, 5%), suggesting a moderate population structure. The result also shows that the CHD spread out wider and the minipig breeds seems to own a subgroup in CHD. Considering that the minipig may endure different breeding goals, we excluded the minipig from CHD in the subsequent analyses.

### Human influence on the demographic history of pigs

**Figure 2:**
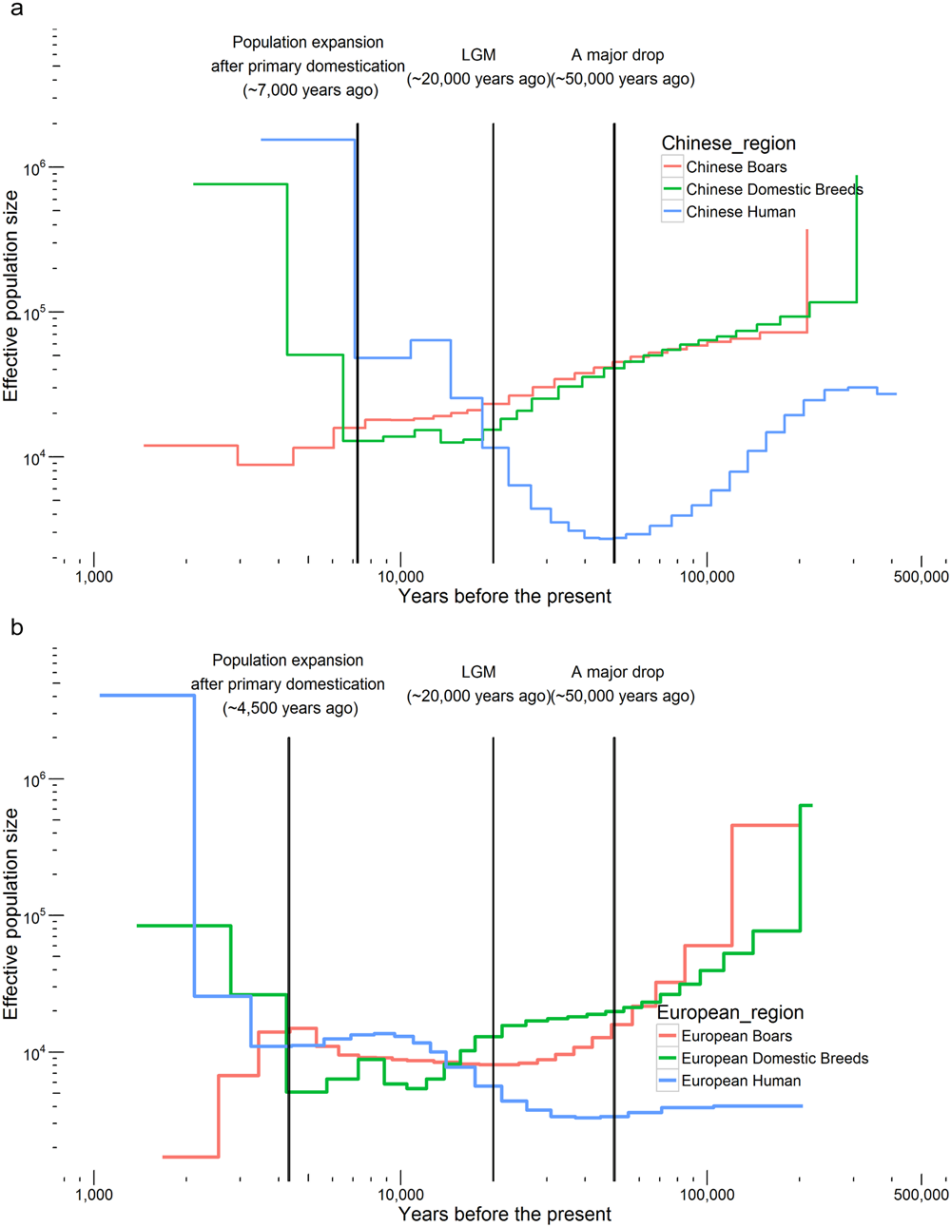
Demographic history of domestic breeds and wild boars in China and Europe. The demographic history of wild boars and domesticate pigs populations was inferred using a hidden Markov model approach as implemented in multiple sequentially Markovian coalescence based on SNP distribution. In the absence of known mutation rates for pig, we used the default mutation rate for human of 2.5 × 10^−8^. For the generation time we used an estimate of 5 years. The data for human’s effective population size refer to previous research^51^. (**a**) Demographic history of domestic breeds and wild boars in Chinese region. 2 individuals were used to indicate the group (2 Rongchang pigs, CHD; 2 North China wild boars, CHW). (**b**) Demographic history of domestic breeds and wild boars in European region. 2 individuals were used to indicate the group (2 Pietrain pigs, EUD; 2 Netherlands wild boars, EUW).

Consistent with previous estimations^21^, we observed a continuous decrease in effective population size for both wild boars and ancestor domesticated pigs from late Quaternary (100,000 BC) to the last glacial maximum (LGM; ~20,000 years ago). This period of decrease is often attributed to cold glacial conditions^21^. However, by comparing demographic histories between pigs and humans, we found that human activity may also influenced pigs’ demography (Fig. 2, Supplementary Figs 2 and 3). Accompanied by a stable increase in human population from late Quaternary (100,000 BC) to the Neolithic Demographic Transition (10,000 BC), both wild boars and the ancestors of domestic pigs experienced a pronounced decline in population size. The association between the increased human effective population size and decreased pig effective population size raises the possibility that human expansion and hunting were important factors in the declining pig population from 10,000–50,000 BC^22^. This hypothesis is supported by a recent study which concluded that human colonization was the dominant driver of ancient megafaunal extinction globally, in addition to climatic factors^23^.

Intriguingly, our demographic analysis identifies distinct trends of the effective population size for wild boars and domestic pigs, starting 4,500–7,000 year ago, which support the conclusion of primary domestication in Europe and China, respectively. Consistent with previous study, there is no evident bottleneck during pig domestication which is likely due to long-term gene flow between domestic pigs and wild boars after domestication^17^. A marked increase in the effective population size of domesticated pigs was observed, while at the same time, the effective population size of wild boars steadily decreased.

Again, these findings are significantly associated with the expansion of the human population, which reflects the fast development of agriculture. We noted a similar pattern was also observed in a recent study of Yak, in which domestic breeds expanded after domestication, while the wild Yak population decreased^24^. Meanwhile, further study is needed to firmly establish the causal relation between agricultural development and the population size of domestic animals.

### Genetic differentiation introduced by human selection

**Figure 3:**
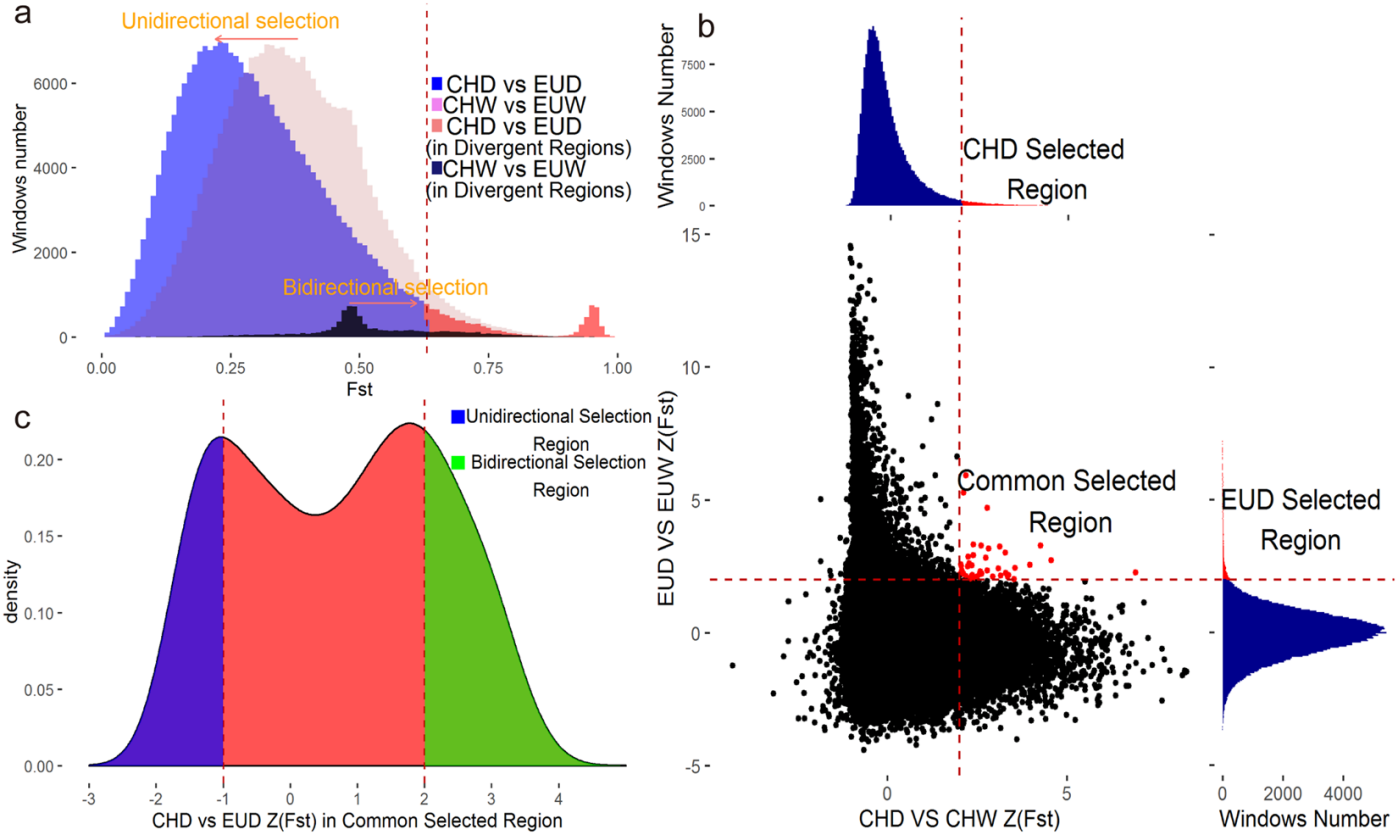
Genomic regions with selection signals during pig domestication. (**a**) Distribution of Fst between each group. The blue region indicated the distribution of CHD vs. EUD’s Fst. The pink region indicated the distribution of CHW vs. EUW’s Fst. The divergent region marked red in this graph is defined as CHD_vs_EUD’s Fst being great than 0.61 (ZFst > 2), and the black part is the distribution of CHW vs. EUW’s Fst in the same region. (**b**) Distribution of EUD vs. EUW’s ZFST and CHD vs. CHWs ZFst, which are calculated in 20-kb windows sliding in 10-kb steps. Data points located to the left and right of the left and right vertical dashed lines, respectively (corresponding to the rights tails of ZFst in CHD vs. CHW and EUD vs. EUW, where the ZFst ratios are both 2), and above the vertical dashed line or horizontal dashed line were identified as selected regions for Chinese domestic pigs and Europe domestic pigs, respectively. The shared selected regions were identified as common selected regions. (**c**) Distribuation of CHD vs. EUD’s ZFst in common selected regions. The right tail marked green correspond to the region with CHD vs. EUD’s ZFst above 2.The left tail marked blue correspond to the region with CHD vs. EUD’s ZFst less than -1.

Previous study showed that domestication involves many gene alleles changing in frequency^25^. We compare genome-wide Wright’s Fst between the Chinese and European population in either wild boars or domestic pigs (Fig. 3a). The overall Fst distribution of CHD vs. EUD has a different shape to that of CHW vs. EUW. The Fst in CHW vs. EUW follow a unimodal distribution while the Fst in CHD vs. EUD show two peaks. We notice that a small peak exists around Fst = 1 in the CHD vs. EUD comparison, indicating that certain genomic regions tend to be extremely divergent. To study what extent the divergence between CHD and EUD are inherited from their corresponding wild counterparts (CHW and EUW), we traced the genes with most significant divergence in CHD vs. EUD comparisons in the Fst distribution of CHW vs. EUW comparisons. As shown in Fig. 3a, the most divergent region between CHD and EUD is not the most divergent between CHW and EUW (marked as black). So, this confirmed that the differences between CHD and EUD were introduced by human-mediated selection during domestication.

To further elucidate the pattern of human-mediated selection, genome-wide scans for selection signatures were performed in both Chinese and European domestic pigs, separately (Fig. 3b). By using a sliding window of 20 kb, we estimated Fst between domestic pigs with their corresponding wild counterparts, and further transformed the Fst value into Z-scores. A threshold of ZFst >2 was then applied to retrieve putative selected regions. Of the approximate 260,000 windows scanned, we selected about 10,000 windows (ZFst > 2) as putative selected regions in CHD and EUD, respectively, comprising 3.8% of the whole genome.

Most of the candidate selected regions were found to be unique in either Chinese or European domestic pigs, with the exception of 313 regions that were shared by CHD and EUD. To ensure the 313 regions constitute real signals, for each region, we performed permutation test by randomly assigning group labels, where sample size in each group were held constant, and re-calculated Fst statistics for 1000 times. 312 out of 313 regions passed the test with p < 0.01. For the 312 candidate common selected regions, interestingly, the ZFst of CHD vs. EUD followed a bimodal distribution with two peaks located at 2 and −1 (Fig. 3c). To investigate regions with clear and reliable features, we thus defined the right part as the candidate bidirectional selection region and the left as the candidate unidirectional selection region.

### Convergent genetic changes in CHD and EUD driven by unidirectional selection

**Figure 4:**
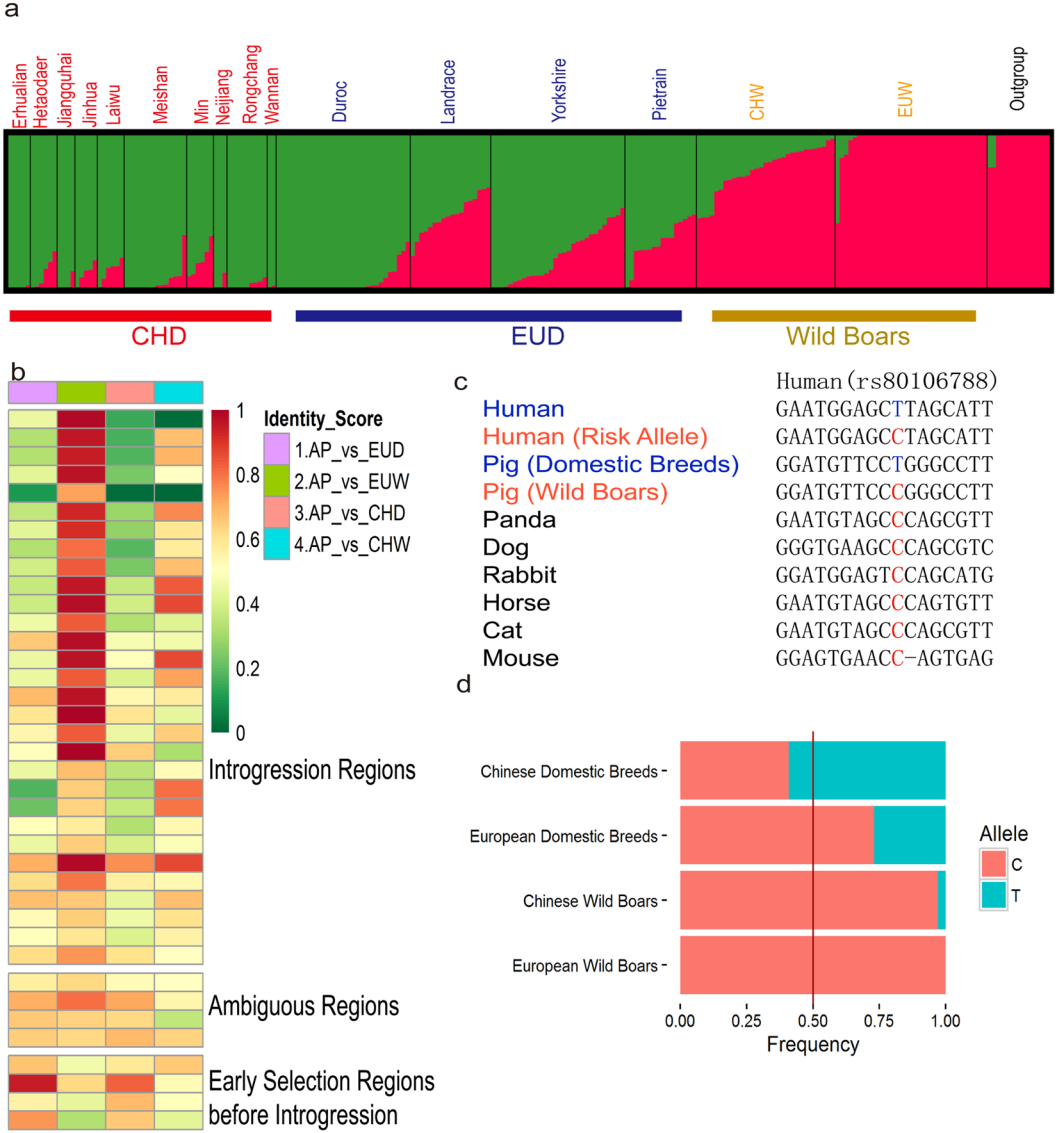
Genomic regions under unidirectional selection and the variant features in *ITPR3*. (**a**) Genetic structure of pig breeds in unidirectional selection region. The length of each colored segment represents the proportion of the individual’s genome from *K* = 2 ancestral populations. The group names are at the top of the figure, and the breed names are at the bottom. (**b**) The heatmap for the identity score of unidirectional selected regions between the ancient pig (AP) and other four groups. SNP frequencies in individual lines were used to calculate identity scores in 20-kb windows. Heat-map colors (right) indicate identity scores. (**c**) The multi-species alignment of human rs80106788 variant in the *ITPR3* gene. (**d**) The allele frequency of the pig rs332886460 in each group.

To avoid false positive due to demographic processes rather than selection, we conducted an additional scan for selective sweeps with the haplotype homozygosity statistic H12 for the 312 regions (See Materials and Methods). By applying a threshold of ZFst CHD vs. EUD’s < −1, H12_CHD_ > H12_CHW_, and H12_EUD_ > H12_EUW_, 39 of the 312 candidate common selected regions were identified as unidirectional selection regions (Supplementary Fig. 4 and Supplementary Table S3). These regions represent the most similar area for CHD and EUD among the entire candidate common selected regions. Population analysis for the unidirectional selection regions demonstrated that, although the degree of transition varied, both Chinese and European domestic pigs exhibited a similar tendency of altering allele frequency from local wild boars (Fig. 4a and Supplementary Fig. 5).

Considering historic introgression between CHD and EUD^26^, the desired alleles in EUD (or CHD) could have either originated directly from EUW (or CHW) or have been introgressed from CHD (or EUD). To distinguish whether the preferentially selected haplotype in EUD originated from the EUW or was introgressed from Chinese breeds, we consulted to an ancient Iberian pig genome. As confirmed by previous study, the ancient pig was clearly domestic and there is no or negligible Asian introgression to Iberian pigs^27^, thus the genotype of the ancient Iberian pig can be used to determine whether the Europe domestic pig had been selected before the introgression from Chinese breeds.

We calculated the identity score (IS) between the ancient pig (AP) and the four defined groups (Fig. 4b). 1 out of the 39 unidirectional selection regions showed no information available due to incomplete genome coverage of the ancient pig. For the remaining 38 regions, we compared the difference between IS_AP vs. CHD_ and IS_AP vs. EUW_. Under the situation that IS_AP vs. CHD_ is larger than IS_AP vs. EUW_, it suggests the ancient pig’s genotype is more similar to Chinese domestic breeds, indicating these regions had been selected in 16 century; if otherwise, the ancient pig’s genotype is more similar to European wild boars than to Chinese domestic breeds, suggesting these regions had not yet been selected in 16 century, but more likely followed an introgression-then-selection process. By investigating the overall differences, we chose 0.1 as the threshold to adjust the difference between IS_AP vs. CHD_ and IS_AP vs. EUW_ (Supplementary Table S4).

**Figure 5:**
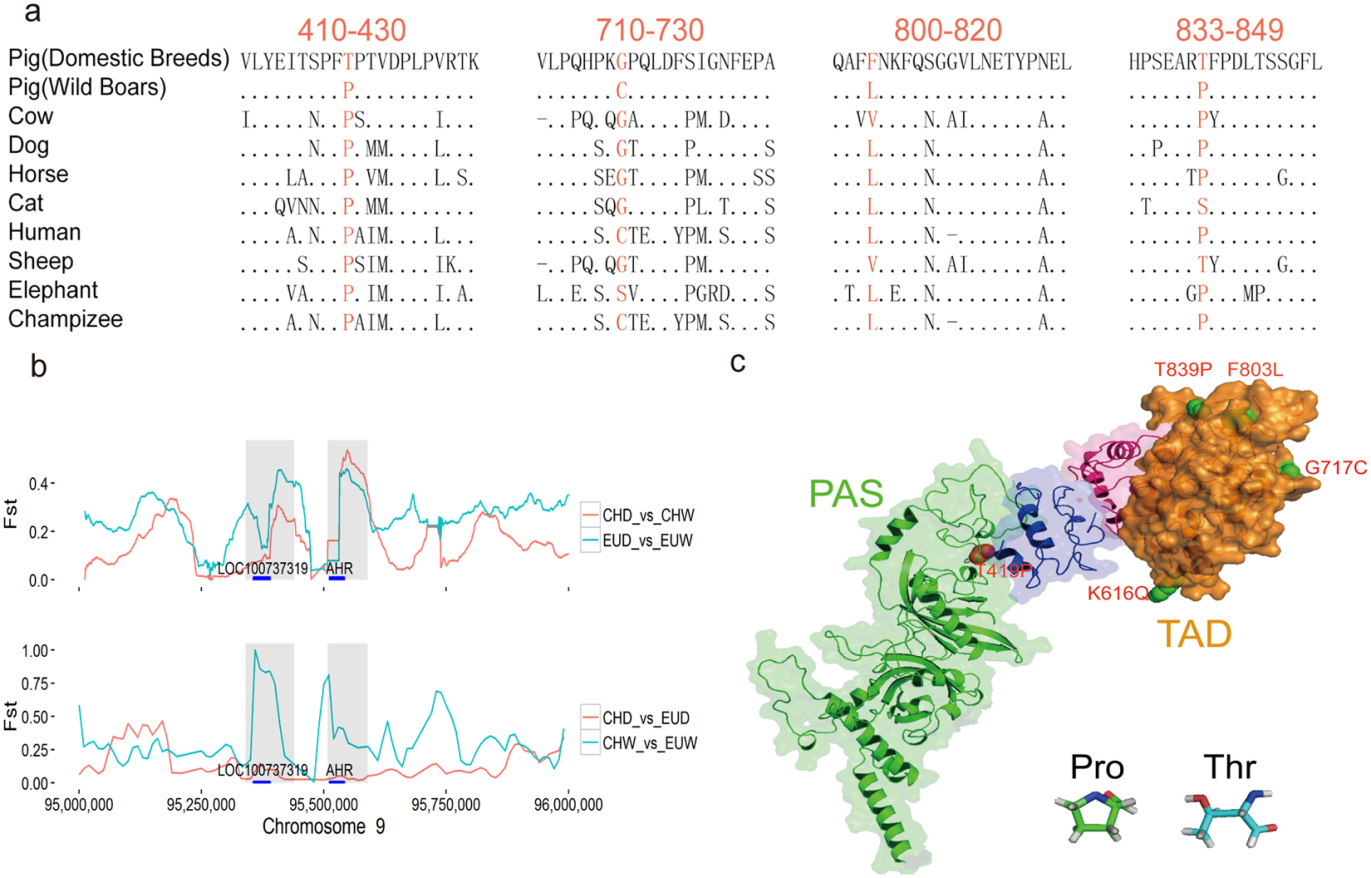
The variants in *AHR* gene and their effects. (**a**) The multi-species alignment of 4 non-synonymous substitutions encoded in the *AHR* gene. Dots indicate identity with the master sequence, and dashes indicate missing data. (**b**) Example of a gene under unidirectional selection pigs. FST values plotted using a 20-kb sliding window. (**c**) Predicted three-dimensional protein structure of AHR. Five non-synonymous substitutions are shown as spheres (P419T, brown; K616Q, G717C, F803L and T839P, green).

As shown in Fig. 5, 30 of the 38 regions showed IS_AP vs. CHD_ < IS_AP vs. EUW + _0.1, suggesting that the selected allele in modern EUD likely origin form Asian introgression. Meanwhile, 4 regions showed IS_AP vs. CHD_ > IS_AP vs. EUW + _0.1, which suggested these regions were more likely to have been selected in 16 century before introgression. That is to say, a large portion of the preferentially selected alleles in EUD was introgressed from Chinese breeds. Similar result was obtained by IBD analysis (Supplementary Fig. 6).

In total, we identified 31 genes overlapping or near all the unidirectional selection regions (Supplementary Table S5). Among the 31 genes, *B3GALT*4, *NR1I2*, *MAATS1*, *PRDM5, LOC100737319* and *AHR* are functionally associated with metabolic diseases including hyperlipidemia, hypertension, and atherosclerosis^28–32^. Compared to the wild boars, the modern domestic pigs could receive adequate food, but had little opportunity for physical activity. Dealing with severe changes of eating habits and living conditions, domestic breeds was selected for metabolism adaptation. Other genes located in the unidirectional selection regions are also functionally plausible bases for the typical domestic features. As an example, we found *MEOX2*, which affect muscle development. The loss of *Meox2* results in smaller limb muscles that harbor reduced numbers of myofibres^33^. We also found *SULT6B1*, *ITPR3*, *KIF25* and *FRMD1* which are associated with the nervous system.

Six genes were found under unidirectional selection in CHD and EUD before Asian introgression, reflecting the prerequisites the in early domestication (Supplementary Table S5). Among these genes, *Dact2* is essential for tooth growth and hair morphology^34, 35^. *NR1I2* determines the behavioral response to xenobiotic^34^. Since these genes were under independent unidirectional selection in CHD and EUD before Asian introgression, suggesting they are important prerequisites for early domestication.

### Unidirectional selection at *ITPR3* locus and *AHR* locus after introgression

Both putative regulatory variants and missense variants were found in the unidirectional selection regions. *ITPR3* plays a key role in exocrine secretion underlying energy metabolism and growth^36^. A mutation (rs332886460) in the intron of *ITPR3* shows frequency differences between wild boars and domestic breeds (Fig. 4c,d). It’s worth noting that the mutation rs332886460 in pigs corresponds to a known variant rs80106788 in human homolog (Fig. 4c,d) which is associated with epilepsy^37^. Selections of the T allele against the C risk allele in both CHD and EUD suggested the common needs on mild temperament during pig domestication.

In addition, *AHR* is a ligand-activated helix-loop-helix transcription factor involved in the regulation of biological responses to planar aromatic hydrocarbons. Previous research identified it as a key factor against fatty liver and hyperlipidemia in humans^38^, and it is suggested to modulate the reproductive process in pigs^26, 39^. Consistent with previous study, we identified nearly absence of genetic differentiation between CHD and EUD and the sharp genetic differentiation between CHW and EUW at the AHR locus.^26^ Notably, five non-synonymous substitutions were identified in the *AHR*, four of which were conserved among vertebrate. This gene was strongly selected in both CHD and EUD, and all five non-substitutions displayed marked allele frequency differences between domestic pigs and wild boars (Fig. 5ab, Supplementary Figs 7 and 8). One substitution, *P419T*, is newly identified in this study. Proline at residue 419 is conserved among all known vertebrate *AHR* sequences. This residue is located in a key position, which connects the Per-AhR/Arnt-Sim (PAS) domain and the trans-activating domain (TAD). Compared to other amino acids, the distinctive cyclic structure of proline’s side chain provides exceptional conformational rigidity, which locks the dihedral angle φ at approximately −65°^40^. Substituting proline with threonine at this position releases the dihedral angle constraint and allows the protein to rotate more freely and to increase its availability for interaction with its co-activator. The other four substitutions were located in exon 11 of the *AHR* gene as previously reported^41^. By modeling the structure of the *AHR* protein using Rosetta (Fig. 5c)^42, 43^, we further show that the remaining four non-synonymous substitutions occur on the surface of the transactivation domain, indicating that they might affect the binding capacity to the co-activators.

### Divergent genetic changes between CHD and EUD driven by bidirectional selection

**Figure 6:**
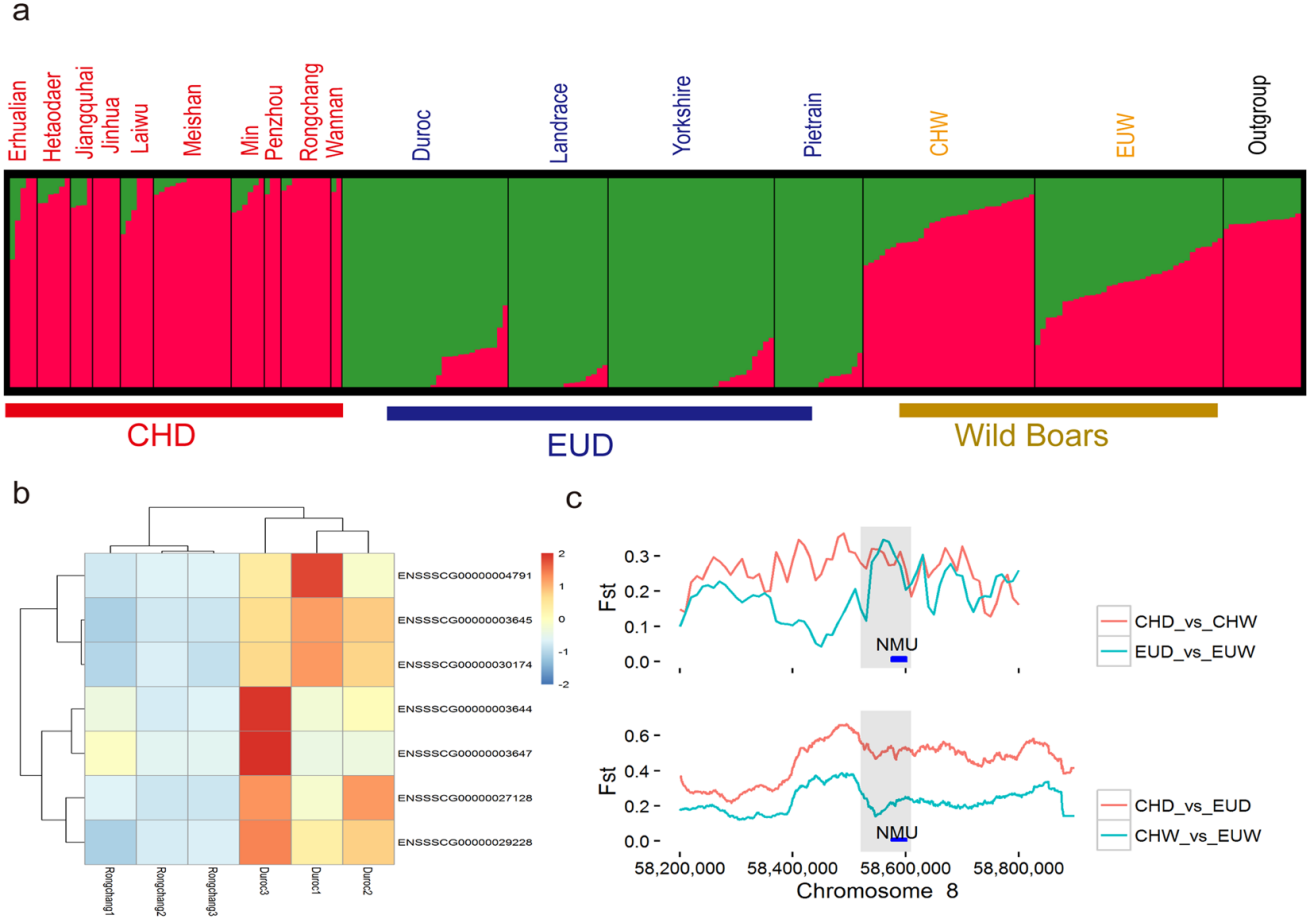
Genomic regions under bidirectional selection and the heatmap for the gene expression of DEG near bidirectional regions in two different breeds. (**a**) Genetic structure of pig breeds in bidirectional selection region. The length of each colored segment represents the proportion of the individual’s genome from K = 2 ancestral populations. The group names are at the top of the figure, and the breed names are at the bottom. (**b**) Heatmap from hierarchical clustering of DEG near bidirectional selection regions. The heatmap was plotted scaled by row. Heat-map colors (right) indicate the relative FPKM. (**c**) Example of a gene under bidirectional selection pigs. FST values plotted using a 20-kb sliding window.

Similarly, for the 312 candidate common selected regions, we applied a threshold of ZFst CHD vs. EUD’s > 2, H12_CHD_ > H12_CHW_, and H12_EUD_ > H12_EUW_ to define bidirectional selection regions. 37 of the 312 regions passed the filtering (Supplementary Fig. 9, Supplementary Table S6). These regions represent the most divergent area for CHD and EUD among the entire candidate common selected regions. For those regions, the CHW and EUW were in a similar heterogeneous state, while the allele frequency of CHD and EUD were nearly fixed in different directions (Fig. 6a and Supplementary Fig. 10).

Bidirectional selection could be driven by distinct selective strategies between Chinese and Europeans. For example, due to a limited source of animal oil and a native diet habit, Chinese traditionally show a strong preference for fatty pork meat, whereas leanness is much more preferred in modern commercial breeds in Europe^26^. Consistent with this, 35 genes near or overlapping the 37 bidirectional selection regions (Supplementary Table S7), there are genes affecting appetite (*NMU*, *CAPS*)^44, 45^, and associated with hyperglycemia and type-2 diabetes (*AP3S2*, *SF3A3*, *ARPIN*, *RPL36*,and *NR3C2*). These findings validate the assertion that the goal of leanness is mainly opposite between Chinese domestic breeds and European commercial breeds in the past^26^.

We also noticed that the genes in bidirectional selection regions belonged predominantly to categories related to reproduction (Chi-squared test, P = 0.0017). Although Chinese domestic pigs exhibit superior reproductive traits compared to European domestic pigs, it is obvious that reproductive capacity would not have been selected against in European domestic pigs. To explain this intriguing result, we propose two possibilities. First, fatty acids and lipids play a key role in various metabolic processes, such as reproduction and embryo development^46, 47^. Intense selection of fat content and leanness could introduce unexpected side effects in other traits, as previously reported^48, 49^. Second, since we found that some genes in the bidirectional selection regions were multifunctional, we speculate that different haplotypes might benefit different traits. For example, *FMNL2* are reported to play roles in the immune system and muscle development^50, 51^. During pig domestication, Europeans prefer to improve leanness in commercial pigs, while Chinese are more concerned about disease resistance. As a result, two haplotypes could have been preferentially selected in CHD and EUD, separately.

As regulatory elements also play important roles in animal domestication^25^, we are expecting that the bidirectional selection regions harbor medium or long range regulatory elements that affect the expression of the genes nearby. Under the premise of that, RNA-seq was applied to analyze global changes in transcriptome from muscle tissue in two different pig breeds (Duroc and Rongchang) (Supplementary Table S8). 953 differently expressed genes (DEG) are identified in the whole genome, with 7 DEG are found being located 20 kb to 250 kb away from the bidirectional region (Fig. 6b, Supplementary Table S9), which is significantly higher than the random expectations for the whole genome (Chi-squared test, P = 0.044). This result suggests that DEGs are more likely affected by the bidirectional selected variants in medium or long range regulatory elements.

### Functional validation of bidirectional selection at the *NMU* locus

**Figure 7:**
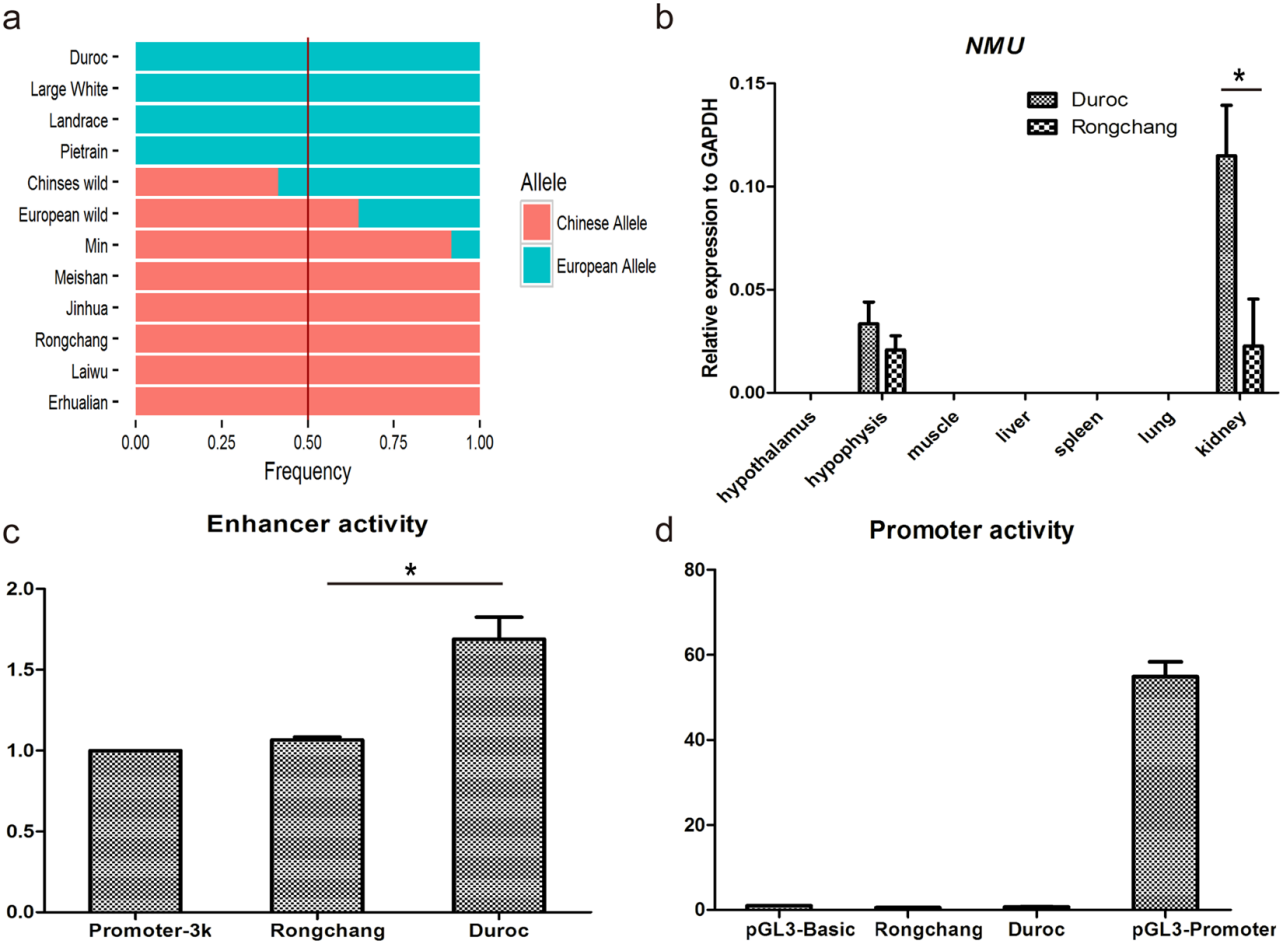
The variants in *NMU* and their effect. (**a**) The allele frequency of the *NMU* in each breed. (**b**) Quantitative RT-PCR Analysis of *NMU* between Rongchang and Duroc in different tissues. *P < 0.05 by Fisher’s exact test. (**c**) Luciferase assay testing the enhancer activity for different allele in the intron of the *NMU* in IBRS-2 cells. (**d**) Luciferase assay testing the promoter activity for different allele in the intron of the *NMU* in IBRS-2 cell.

The *NMU* (*Neuromedin U*) gene has been identified as a hypothalamic neuropeptide that regulates body weight and fat mass^52^. *NMU* knockout mice exhibited increased body weight and adiposity, hyperphagia, and decreased locomotor activity and energy expenditure^44^. In addition, multiple polymorphisms in the intron of *NMU* are reported to be associated with metabolic disease and type-2 diabetes in humans^53^. We confirmed that the genomic region around *NMU* is under bidirectional selection between CHD vs. EUD with multiple test statistics (Figs 6c and 7a, and Supplementary Fig. 11).

By analyzing the variants with high allele frequency difference (ΔAF) between CHD and EUD, we determined that the location of the differential region was at the second intron of *NMU*. Meanwhile, according to the ENCODE data of the homologous regions in humans, this region was suggested for a potential regulatory role.

In order to confirm its biological relevance, we quantified the expression level of *NMU* in CHD and EUD in various tissues of newborn piglets, using qRT-PCR. As shown in Fig. 7b, the expression of *NMU* was observed in hypophysis and kidney, with a statistical difference found in hypophysis and kidney tissues between CHD and EUD, suggesting potential enhancer activities for the intron sequence.

Multiple enhancer and promoter Luciferase vectors were further constructed to investigate the putative effect of the variants on the expression of *NMU*. We transferred the vectors into IBRS-2 cells and fibroblast cells to evaluate the function of the variants. The results of the Luciferase assay in IBRS-2 cells and fibroblast cells were similar. The European haplotype was of higher enhancer activity than that of Chinese haplotype, but neither exhibited promoter activity (Fig. 7c,d, and Supplementary Fig. 12). Furthermore, the European allele showed higher activity than the Chinese allele. Intriguingly, a previous study demonstrated that CHD exhibited a higher *NMU* expression level in pregnant placenta than EUD^54^. It seems that the intron region contributes the differential expression between CHD and EUD in a tissue-specific manner.

## Discussion

In this study, we aimed to investigate how human preferences profoundly altered the traits of domestic breeds of pigs. We found that unidirectional selection and bidirectional selection laid the foundation for the convergent and divergent changes in domestic breeds.

Preferences in domestication are largely shared among Chinese and European. Common breeding goals in Chinese and European domestic breeds brings convergent genetic changes, involving genes mostly related to the nervous system, muscle development, and especially to metabolic diseases. And these genes reflect key traits in pig domestication, including docile temperaments, rapid growth and especially the metabolism adaptation to the fattening lifestyle. Consist with previous research, most convergent change result from unidirectional selection after introgression^26^. What is more, as the CHD and CHW show strong signatures of selection in these regions, it suggest that the Chinese domestic breeds had been selected for desire traits in the first place, then the preferred alleles was passed to EUD by introgression, and then the preferred allele were also selected in EUD.

On the other hand, different or even contrasting domestication preference will lead to distinct features of animals between different domestication centers. Chinese prioritize fat deposits and reproduction while European preferred leanness and body length for modern commercial breeds. To achieve different breeding goals, distinct haplotypes were preferentially selected and fixed in opposite directions, which ultimately results in divergent genetics changes contributing distinct leanness, disease resistance, and reproductive traits between CHD and EUD.

Since selection preferentially acts on loci with major effects during domestication^55^, if one locus was selected in CHD and EUD simultaneously, it is more likely that the loci would contribute a large effect on the desirable trait. It’s worth noting that sizable genes, either unidirectional selected or bidirectional selected, are associated with metabolism adaption or obesity. Thus research on these genes could provide insight to human metabolism disease.

In addition, our demographic analyses suggest that human activities may have a longer and broader impact on pigs than was previously thought. Human expansion and hunting could have contributed to the decline of pigs in the pre-domestication period (50,000 BP). Furthermore, accompanied by the rapid growth of the human population, the beginning of both CHD and EUD pig domestication experienced a rapidly expanding with the development of agriculture. Intriguingly, Chinese breeds show this expansion earlier (approximately 7,000 years ago) than European domestic breeds (approximately 4,500 years ago), consistent with historical records that imply that the extensive domestication of pigs may occurred earlier in China^1, 5^.

In conclusion, in this study, we demonstrated the role of human activities and human-mediated selection on pig domestication. In the long-term coevolution between humans and pigs, similar and diverse needs between Chinese and Europeans led convergent and divergent genetic changes in domestic breeds. Our results will help to elucidate the significant human impacts that occurred during pre-domestication and the domestication process, as well as facilitate future studies of gene functions for domestication.

## Materials and Methods

### Ethics statements

This study was approved by the Animal Welfare Committee of China Agricultural University. The approval number is SKLAB-2012-11. All pigs used in this study were taken care and operated according to the relevant regulations.

### Genome resequencing and SNP calling

We sampled eight pigs, including four Rongchang pigs and four Duroc pigs. Genomic DNA was extracted from the ear tissues of each individual. Sequencing was performed on the Illumina HiSeq. 2500 platform. In addition, we downloaded the genome data of 249 individuals across the world from the EMBL database^4, 10, 18, 21, 56^, including 93 European domestic pigs, 77 domestic pigs in China, 31 Asian wild boars, 34 European wild boars, and 14 individuals representing 7 other species (Supplementary Table S1).

Filtered reads from all individuals were aligned to the susScr3 reference genome by Bowtie2 with default settings^57^. Then, the Genome Analysis Toolkit (GATK) 3.2.2 was utilized for SNP calling for each groups following the GATK best practice pipeline^58^. To exclude potential false positive variant calls, we applied variant quality recalibration by GATK, using the command ‘VariantFiltration’ with the parameters ‘–filterExpression “QD < 10.0||MQ < 40.0||FS > 60.0||ReadPosRankSum < − 8.0” −G_filter “GQ < 20”.

### Population analysis

Principal component analysis (PCA) was conducted using PLINK v.107 and GCTA^59, 60^ based on the all 94 million SNP. Identity-by-state (IBS) distance matrix for all the individuals was built by PLINK v.107 and R package dendextend^61^. The population structure in selected regions was determined by ADMIXTURE software^62^.

### Demographic history reconstruction

The demographic history of wild boar and domesticated pig populations was inferred using a hidden Markov model approach, as implemented in multiple sequentially Markovian coalescence (MSMC version 2.0) based on SNP distribution^63^. Both phased data and unphased data are used to infer demographic history, respectively. For phased data, the individuals were phased against a inferred reference panel of the sub-breeds by Beagle version 3.3.2. The most recent windows are excluded from the results due to low credibility according to the previous research^64^. We used the mutation rate of 2.5 × 10^–8^ and a generation time of 5 years referring to previous research. Results from phased data are shown in Fig. 2 and Supplementary Fig. 2, while the results from unphased data are shown in Supplementary Fig. 3. Although they are different in details, the phased results and unphased results have similar trends to support our opinions.

### Detection of selection signals

We applied both allele frequency-based and haplotype-based statistics to detect selection. First, a sliding-window approach (20-kb windows sliding in 10-kb steps) was applied to quantify genetic differentiation (FST) by vcftools^65^ between each group. Based on the Fst under the null hypothesis of no differences between the domestic and wild groups, an empirical p value was estimated as P = (n + 1)/1001, where n was the counts of the permutated sets for which the Fst was equal to or greater than the observed Fst in the real data. We calculated absolute allele frequency difference (ΔAF) and Tajima’s D were calculated to confirm the selection signals by vcftools^65^.

Then we introduce H12, a haplotype-based statistical test capable of detecting both hard and soft sweeps. H12 quantifies haplotype homozygosity after combining the two most frequent haplotypes into one class so that H12 = (*p*_1_ + ${p}_{2}^{\,}$)^2^ + $\sum _{i > 2}\,{p}_{i}^{2}$, where ${p}_{i}^{\,}$ is the frequency of the $i\,$th haplotype in a window of fixed size^66^. H12 was calculated for each group using SelectionHapStats software as described by Nandita R. Garud^67^. Analysis window size (in terms of SNPs) is 50, and distance between the centers of analysis windows (in terms of SNPs) is 10. To avoid the bias on H12 value of each group brought by different sample size, we random select the same number of individuals in domestic groups that are used in the wild groups for ten times. H12 value for domestic groups was the average of the results from the ten runs.

### Calculation of identity scores

We calculated identity scores (IS)^68^, to visualize haplotype sharing in pairwise comparisons in the unidirectional selection regions. For each identified SNP, we determined the frequency of SNPs that corresponded to the reference allele, in each groups. The IS values of groups’ SNPs were then calculated as IS_Group1 vs. Group2_ = 1 − (|F_Group1_ − F_Group2_|), with positions assessed only if at least half individuals was obtained in each groups. The IS value for a window was averaged for all SNPs in the window.

### Pairwise IBD detection

The genome was divided into bins of 20,000 bp and the IBD detection was done by Beagle version 3.3.2 as described by Mirte Bosse *et al*.^26, 69^. The threshold used for IBD detection was 5.0^−6^. Normalized IBD for Group1 vs. Group2: nIBD = cIBD/tIBD (where cIBD = count of all haplotypes IBD between Group1 and Group2 and tIBD = total pairwise comparisons between Group1 and Group2).

### Reconstruction of the protein structure of *AHR*

The structure of *AHR* was constructed by Rosseta^70^. Both comparative and *de novo* strategy were performed to construct the structure.

### Identification of DEG between Duroc and Rongchang pig in muscle tissue

To investigate the DEG between Duroc and Rongchang pig in muscle tissue, RNA-Seq analysis was performed on six piglets representing two breeds: Duroc and Ronghcang (three animals of each breed). RNA was extracted from the longissimus dorsi of each individual. Sequencing was performed on the Illumina HiSeq. 2500 platform. Since Rongchang is divergent from Duroc, the regions with too many differences in the genome will reduce the efficacy of accuracy of reads mapping to the genome, and in turn, will cause improper estimating of the transcript level. In order to eliminate the mapping bias, the pseudogenomes of Duroc and Rongchang pig were thus created respectively by replacing the reference allele with variant allele matching each breeds (Duroc and Rongchang). Then, the clean reads were again mapped to the corresponding pseudogenome using Hisat2^71^. Differentially expressed genes are identified by CUFFLINKS^72^.

### Quantitative RT-PCR analysis of *NMU* in different tissues

Total RNA was isolated from 3 Rongchang and 3 Duroc new born piglets with RNeasy Fibrous Tissue Mini Kit (Qiagen) according to the manufacturer’s protocol. Synthesis of cDNA was performed by M-MLV reverse transcriptase (Promega). We used LightCycler 480 SYBR Green I Master (Roche) to prepare the PCR mix, and the quantitative RT-PCR was performed on LightCycler^®^ 480 (Roche) following the program: pre-incubation (95 °C, 5 min), amplification (95 °C, 10 s; 60 °C, 10 s; 72 °C, 10 s) 30 cycles, melting curve (95 °C, 5 s; 65 °C, 1 min), cooling (40 °C, 10 s). Gene expression was normalized to the GAPDH. The primers used in this experiment are shown in Supplementary Table S10.

### Enhancer activity detection

The partial sequence of *NMU* intron 2 were amplified from Rongchang and Duroc genomic DNA, respectively, and then subcloned into pGL3-Promoter Vector (Promega) at the Nhe I/Bgl II restriction sites, which lie upstream of the SV40 promoter driving the luciferase reporter gene. The primer pairs NMU-F/NMU-R are shown in Supplementary Table S10. Unexpectedly, we found that the sequence provided in NCBI was inconsistent with our results based on Sanger sequencing. The sequence supplied by the NCBI contains an extra repeat sequence (Chr8: 58585019-58585904), which is approximately 900 bp. Then, we selected multiple breeds, including Duroc, Landrace, Rongchang pig and Xiang pig, to confirm that the extra repeat sequence does not exist, and that it is more likely that the sequence provided in the NCBI was incorrect. The sequences cloned from Duroc and Rongchang are supplied in Supplementary Table S11.

Cotransfection of pGL3-NMUPromoter Vector and internal control vector pRL-TK (Promega) was performed into IBRS-2 cells mediated by Lipofectamine^®^ 3000 transfection reagent (Invitrogen). Cells in 70–90% confluent were co-transfected in 24-well plates. Each transfection contained 0.45 µg pGL3-Promoter Vector, 0.05 µg pRL-TK, 1 µl P3000TM reagent, and 1.5 µl Lipofectamine^®^ 3000 reagent. After 48 h of transfection, cells underwent dual-luciferase reporter assay, following the Dual-Glo^®^ Luciferase Assay System (Promega) protocol. We firstly measured the firefly luminescence of pGL3-Promoter Vector, and then measured the Renilla luminescence of pRL-TK. The relative luciferase activity was calculated as the ratio from firefly luminescence to Renilla luminescence. We took pGL3-Promoter-3k Vector (Promega), which has 3 kb random sequence (no enhancer activity) at the Nhe I/Bgl II restriction sites, to promote the expression of luciferase reporter gene, as a control (Supplementary Table S11). Each experiment was repeated three times. The same experiment was performed in pig fetal fibroblasts.

### Promoter activity detection

The 3.19 kb Rongchang and Duroc *NMU* sequences were subcloned into pGL3-Basic Vector (Promega), which has no promoter upstream of luciferase reporter gene, at the Nhe I/Bgl II restriction sites, respectively. Cotransfection of pGL3-NMUbasic-Vector and internal control vector pRL-TK (Promega) was performed into IBRS-2 cells mediated by Lipofectamine^®^ 3000 transfection reagent (Invitrogen). We took pGL3-Basic Vector as a negative control and took pGL3-Promoter Vector as a positive control. After 48 h of transfection, cells underwent dual-luciferase reporter assay. We calculated the relative luciferase activity as upside in enhancer activity detection. Each experiment was repeated three times. The same experiment was performed in pig fetal fibroblasts.

## Electronic supplementary material

Supplementary_Figures

Supplementary_Tables

## References

[1] Ramos-Onsins SE, Burgos-Paz W, Manunza A, Amills M (2014). Mining the pig genome to investigate the domestication process. Heredity (Edinb).

[2] Larson G (2005). Worldwide phylogeography of wild boar reveals multiple centers of pig domestication. Science.

[3] Cucchi T (2011). A. H.-B., J. Yuan,K. Dobney. Early Neolithic pig domestication at Jiahu, Henan Province, China: clues from molar shape analyses using geometric morphometric approaches. Journal of Archaeological Science.

[4] Frantz, L. A. *et al*. Evidence of long-term gene flow and selection during domestication from analyses of Eurasian wild and domestic pig genomes. *Nature genetics*, doi:10.1038/ng.3394 (2015). 10.1038/ng.3394 26323058

[5] Larson G (2007). Ancient DNA, pig domestication, and the spread of the Neolithic into Europe. Proceedings of the National Academy of Sciences of the United States of America.

[6] Ottoni C (2013). Pig domestication and human-mediated dispersal in western Eurasia revealed through ancient DNA and geometric morphometrics. Molecular biology and evolution.

[7] Rubin CJ (2012). Strong signatures of selection in the domestic pig genome. Proceedings of the National Academy of Sciences of the United States of America.

[8] Wilkinson S (2013). Signatures of diversifying selection in European pig breeds. PLoS genetics.

[9] Amaral AJ (2011). Genome-wide footprints of pig domestication and selection revealed through massive parallel sequencing of pooled DNA. PloS one.

[10] Kim H (2015). Exploring the genetic signature of body size in Yucatan miniature pig. PloS one.

[11] Groenen MA (2016). A decade of pig genome sequencing: a window on pig domestication and evolution. Genetics, selection, evolution: GSE.

[12] Jeong H (2015). Exploring evidence of positive selection reveals genetic basis of meat quality traits in Berkshire pigs through whole genome sequencing. BMC genetics.

[13] Yang J (2016). Whole-Genome Sequencing of Native Sheep Provides Insights into Rapid Adaptations to Extreme Environments. Molecular biology and evolution.

[14] Wang C (2015). Genome-wide analysis reveals artificial selection on coat colour and reproductive traits in Chinese domestic pigs. Molecular ecology resources.

[15] Van Laere AS (2003). A regulatory mutation in IGF2 causes a major QTL effect on muscle growth in the pig. Nature.

[16] Moon S (2015). A genome-wide scan for signatures of directional selection in domesticated pigs. BMC genomics.

[17] Frantz LA (2015). Evidence of long-term gene flow and selection during domestication from analyses of Eurasian wild and domestic pig genomes. Nature genetics.

[18] Li M (2013). Genomic analyses identify distinct patterns of selection in domesticated pigs and Tibetan wild boars. Nature genetics.

[19] Gerstein HC, Waltman L (2006). Why don’t pigs get diabetes? Explanations for variations in diabetes susceptibility in human populations living in a diabetogenic environment. CMAJ: Canadian Medical Association journal=journal de l’Association medicale canadienne.

[20] Dyson MC, Alloosh M, Vuchetich JP, Mokelke EA, Sturek M (2006). Components of metabolic syndrome and coronary artery disease in female Ossabaw swine fed excess atherogenic diet. Comparative medicine.

[21] Groenen MA (2012). Analyses of pig genomes provide insight into porcine demography and evolution. Nature.

[22] Stanyon R, Sazzini M, Luiselli D (2009). Timing the first human migration into eastern Asia. Journal of biology.

[23] Bartlett LJ, Graham DRW (2015). W. Prescott2, Andrew Balmford2, Rhys E. Green2,3, Anders Eriksson2, Paul J. Valdes4, Joy S. Singarayer5 andAndrea Manica. Robustness despite uncertainty: regional climate data reveal the dominant role of humans in explaining global extinctions of Late Quaternary megafauna. Ecography.

[24] Qiu Q (2015). Yak whole-genome resequencing reveals domestication signatures and prehistoric population expansions. Nature communications.

[25] Carneiro M (2014). Rabbit genome analysis reveals a polygenic basis for phenotypic change during domestication. Science.

[26] Bosse M (2014). Genomic analysis reveals selection for Asian genes in European pigs following human-mediated introgression. Nature communications.

[27] Ramirez O (2015). Genome data from a sixteenth century pig illuminate modern breed relationships. Heredity.

[28] Willer CJ (2008). Newly identified loci that influence lipid concentrations and risk of coronary artery disease. Nature genetics.

[29] Sui Y (2015). Intestinal pregnane X receptor links xenobiotic exposure and hypercholesterolemia. Molecular endocrinology.

[30] Talmud PJ (2003). Progression of atherosclerosis is associated with variation in the alpha1-antitrypsin gene. Arteriosclerosis, thrombosis, and vascular biology.

[31] Huang S (2015). AhR expression and polymorphisms are associated with risk of coronary arterial disease in Chinese population. Scientific reports.

[32] Meigs JB (2007). Genome-wide association with diabetes-related traits in the Framingham Heart Study. BMC medical genetics.

[33] Otto A (2010). A hypoplastic model of skeletal muscle development displaying reduced foetal myoblast cell numbers, increased oxidative myofibres and improved specific tension capacity. Developmental biology.

[34] Bult CJ (2015). Mouse Tumor Biology (MTB): a database of mouse models for human cancer. Nucleic acids research.

[35] Li X, Florez S, Wang J, Cao H, Amendt BA (2013). Dact2 represses PITX2 transcriptional activation and cell proliferation through Wnt/beta-catenin signaling during odontogenesis. PloS one.

[36] Futatsugi A (2005). IP3 receptor types 2 and 3 mediate exocrine secretion underlying energy metabolism. Science.

[37] Speed D (2014). A genome-wide association study and biological pathway analysis of epilepsy prognosis in a prospective cohort of newly treated epilepsy. Human molecular genetics.

[38] Xiao L, Zhang Z, Luo X (2014). Roles of xenobiotic receptors in vascular pathophysiology. Circulation journal: official journal of the Japanese Circulation Society.

[39] Jablonska O (2013). C. R. The expression of aryl hydrocarbon receptor in porcine ovarian cells. Reprod Domest Anim.

[40] Morris AL, MacArthur MW, Hutchinson EG, Thornton JM (1992). Stereochemical quality of protein structure coordinates. Proteins.

[41] Harper PA, Wong J, Lam MS, Okey AB (2002). Polymorphisms in the human AH receptor. Chemico-biological interactions.

[42] Bradley P, Misura KM, Baker D (2005). Toward high-resolution de novo structure prediction for small proteins. Science.

[43] Simons KT, Kooperberg C, Huang E, Baker D (1997). Assembly of protein tertiary structures from fragments with similar local sequences using simulated annealing and Bayesian scoring functions. Journal of molecular biology.

[44] Hanada R (2004). Neuromedin U has a novel anorexigenic effect independent of the leptin signaling pathway. Nature medicine.

[45] Pinheiro, A. P. *et al*. Association study of 182 candidate genes in anorexia nervosa. American journal of medical genetics. Part B, Neuropsychiatric genetics: the official publication of the International Society of Psychiatric Genetics **153B**, 1070–1080, doi:10.1002/ajmg.b.31082 (2010). 10.1002/ajmg.b.31082 PMC2963154 20468064

[46] Cao S (2014). Specific gene-regulation networks during the pre-implantation development of the pig embryo as revealed by deep sequencing. BMC genomics.

[47] Hansen M, Flatt T, Aguilaniu H (2013). Reproduction, fat metabolism, and life span: what is the connection?. Cell metabolism.

[48] Fernandez-Marin B (2014). Side-effects of domestication: cultivated legume seeds contain similar tocopherols and fatty acids but less carotenoids than their wild counterparts. BMC plant biology.

[49] Rauw WM, Kanis E, Noordhuizen-Stassen EN, Grommers FJ (1998). Undesirable side effects of selection for high production efficiency in farm animals: a review. Livestock Production Science.

[50] Rosado M (2014). Critical roles for multiple formins during cardiac myofibril development and repair. Mol Biol Cell.

[51] Lauc G (2013). Loci associated with N-glycosylation of human immunoglobulin G show pleiotropy with autoimmune diseases and haematological cancers. PLoS Genet.

[52] Hainerova I (2006). Association between neuromedin U gene variants and overweight and obesity. The Journal of clinical endocrinology and metabolism.

[53] Dastani Z (2012). Novel loci for adiponectin levels and their influence on type 2 diabetes and metabolic traits: a multi-ethnic meta-analysis of 45,891 individuals. PLoS genetics.

[54] Gu T (2014). Endometrial gene expression profiling in pregnant Meishan and Yorkshire pigs on day 12 of gestation. BMC genomics.

[55] Wright D (2015). The Genetic Architecture of Domestication in Animals. Bioinformatics and biology insights.

[56] Ai H (2015). Adaptation and possible ancient interspecies introgression in pigs identified by whole-genome sequencing. Nature genetics.

[57] Langmead B, Salzberg SL (2012). Fast gapped-read alignment with Bowtie 2. Nature methods.

[58] McKenna A (2010). The Genome Analysis Toolkit: a MapReduce framework for analyzing next-generation DNA sequencing data. Genome research.

[59] Purcell S (2007). PLINK: a tool set for whole-genome association and population-based linkage analyses. American journal of human genetics.

[60] Yang J, Lee SH, Goddard ME, Visscher PM (2011). GCTA: a tool for genome-wide complex trait analysis. American journal of human genetics.

[61] Galili T (2015). dendextend: an R package for visualizing, adjusting and comparing trees of hierarchical clustering. Bioinformatics.

[62] Alexander DH, Novembre J, Lange K (2009). Fast model-based estimation of ancestry in unrelated individuals. Genome research.

[63] Schiffels S, Durbin R (2014). Inferring human population size and separation history from multiple genome sequences. Nature genetics.

[64] Boitard S, Rodriguez W, Jay F, Mona S, Austerlitz F (2016). Inferring Population Size History from Large Samples of Genome-Wide Molecular Data - An Approximate Bayesian Computation Approach. PLoS genetics.

[65] Danecek P (2011). The variant call format and VCFtools. Bioinformatics.

[66] Crisci JL, Dean MD, Ralph P (2016). Adaptation in isolated populations: when does it happen and when can we tell?. Molecular ecology.

[67] Garud NR, Messer PW, Buzbas EO, Petrov DA (2015). Recent selective sweeps in North American Drosophila melanogaster show signatures of soft sweeps. PLoS genetics.

[68] Rubin CJ (2010). Whole-genome resequencing reveals loci under selection during chicken domestication. Nature.

[69] Browning BL, Browning SR (2011). A fast, powerful method for detecting identity by descent. American journal of human genetics.

[70] Kim DE, Chivian D, Baker D (2004). Protein structure prediction and analysis using the Robetta server. Nucleic acids research.

[71] Kim D, Langmead B, Salzberg SL (2015). HISAT: a fast spliced aligner with low memory requirements. Nature methods.

[72] Trapnell C (2012). Differential gene and transcript expression analysis of RNA-seq experiments with TopHat and Cufflinks. Nature protocols.

