# Supplementary material for: Convergent and divergent genetic changes in the genome of Chinese and European pigs
Source: Sci Rep. 2017 Aug 17;7:8662. doi: 10.1038/s41598-017-09061-z (PMC5561219; doi:10.1038/s41598-017-09061-z)
Supplement: Supplementary file 1 — Supplementary_Figures [file 41598_2017_9061_MOESM1_ESM.pdf]

Convergent and divergent genetic changes in the genome of Chinese and European pigs

Jing Wang[1,2+], Huiying Zou[2,7+], Lei Chen[3], Xi Long[3], Jing Lan[3], Wenjie Liu[2], Li Ma[2], Chao Wang[4], Xinyu Xu[5], Liming Ren[2], Zhenglong Gu[6], Ning Li[2], Xiaoxiang Hu[2], Yaofeng Zhao[2]* & Yiqiang Zhao[1,2]*.

Supplemental Information

Supplementary Figures S1-S12

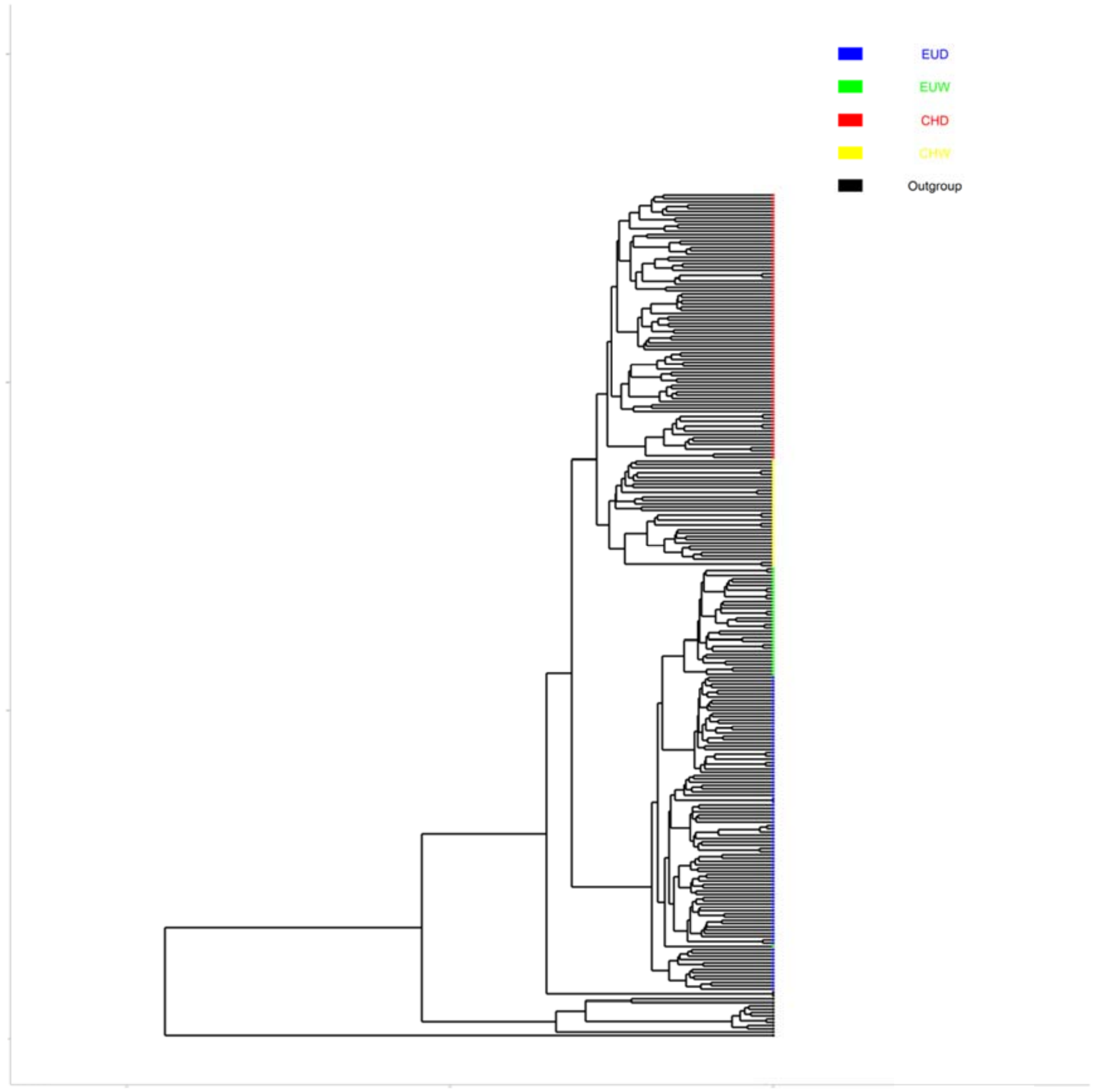

Supplementary Figure 1. Neighbor-joining tree in whole genome regions. Neighbor-joining tree is based on an identity-by-state (IBS) distance matrix, using the data in the whole genome regions. The CHD include Mini-pigs.

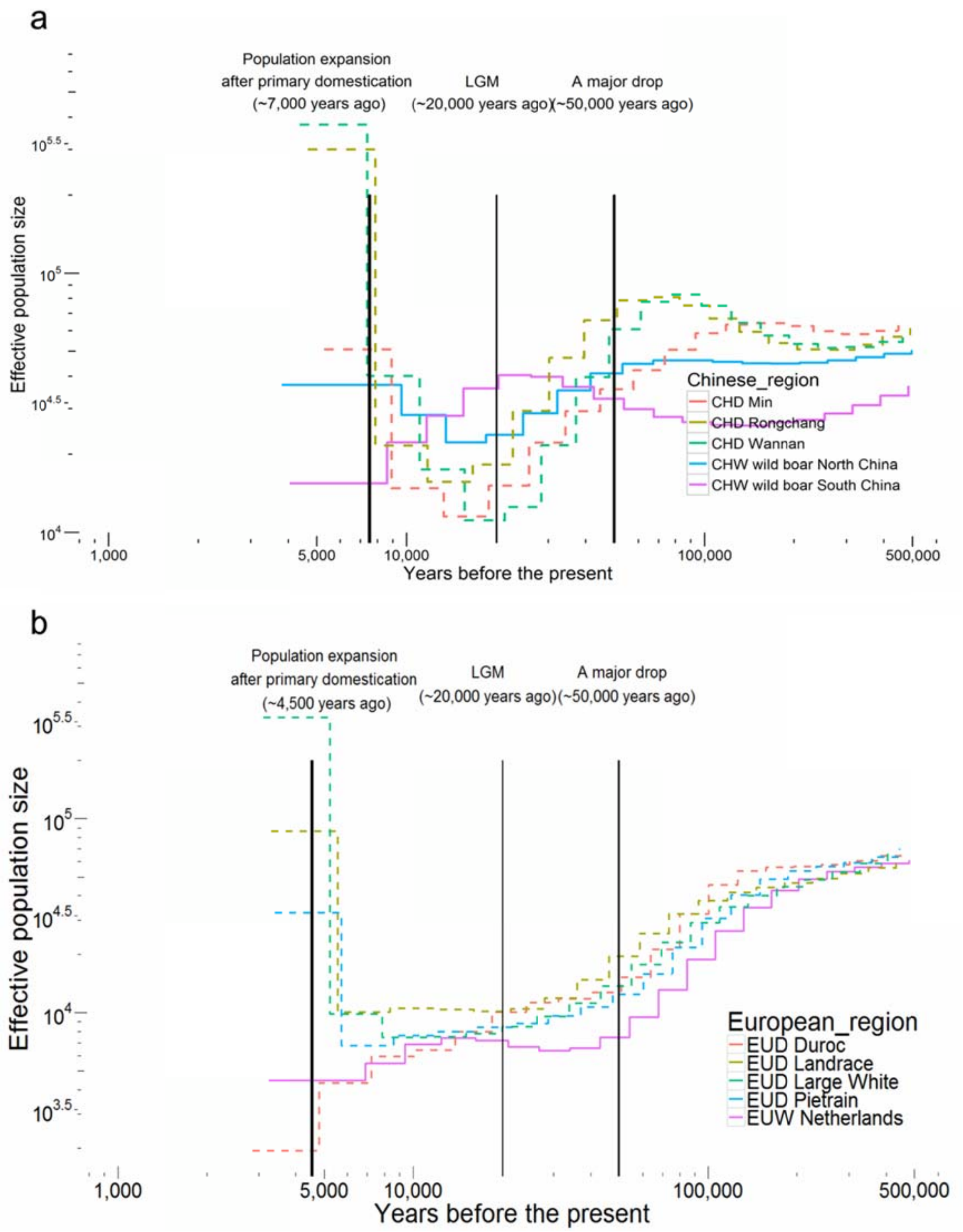

Supplementary Figure 2. Demographic history of domestic breeds and wild boars in China and Europe on phased data. 2 individuals from each breeds were used to infer the demographic history using a hidden Markov model approach as implemented in multiple sequentially Markovian coalescence based on SNP distribution. The Demographic history is inferred based on phased data. **(a),** Demographic history of domestic breeds and wild boars in China. **(b),** Demographic history of domestic breeds and wild boars in Europe.

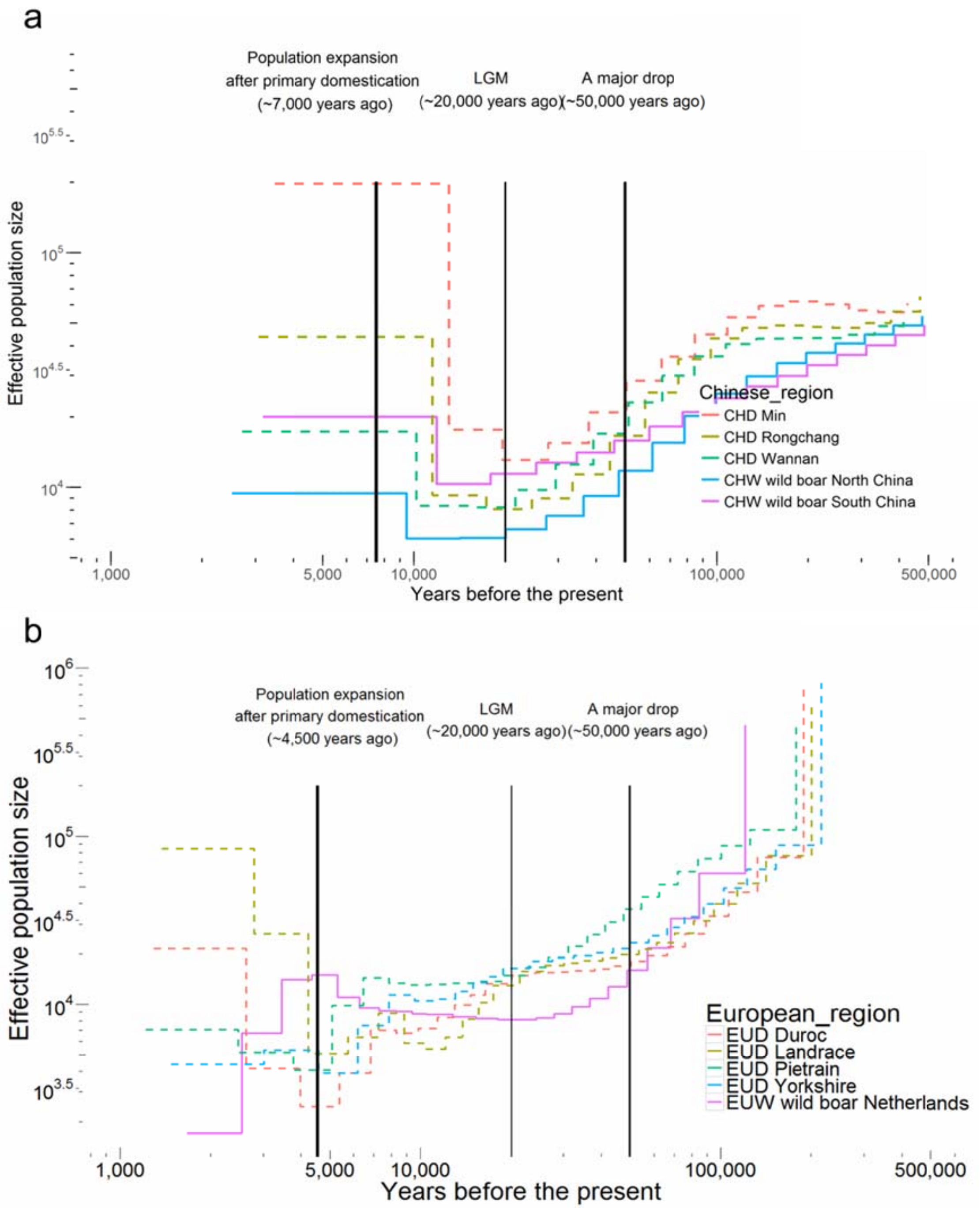

Supplementary Figure 3. Demographic history of domestic breeds and wild boars in China and Europe on unphased data. 2 individuals from each breeds were used to infer the demographic history using a hidden Markov model approach as implemented in multiple sequentially Markovian coalescence based on SNP distribution. The Demographic history is inferred based on unphased data. **(a),** Demographic history of domestic breeds and wild boars in China. **(b),** Demographic history of domestic breeds and wild boars in Europe.

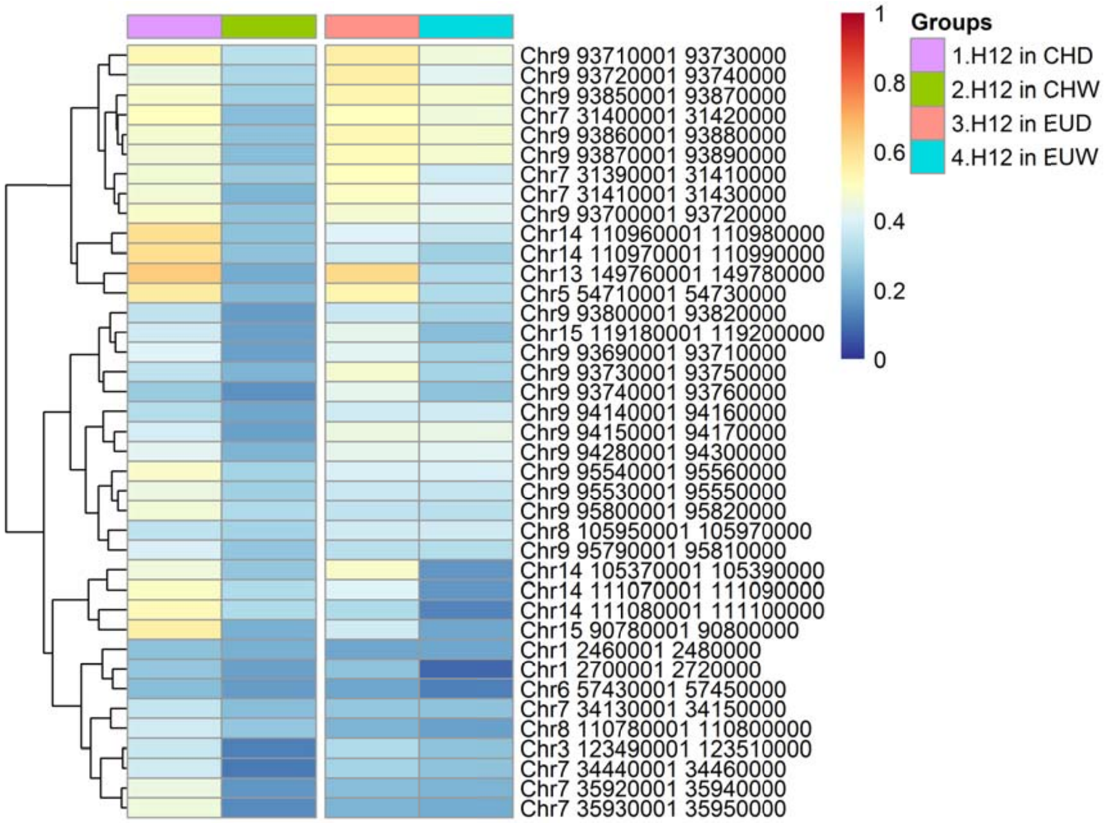

Supplementary Figure 4. The H12 in unidirectional selection regions for each group. Heatmap from hierarchical clustering of the H12 value in the 39 unidirectional regions for each group. Heat-map colors (right) indicate the H12 value.

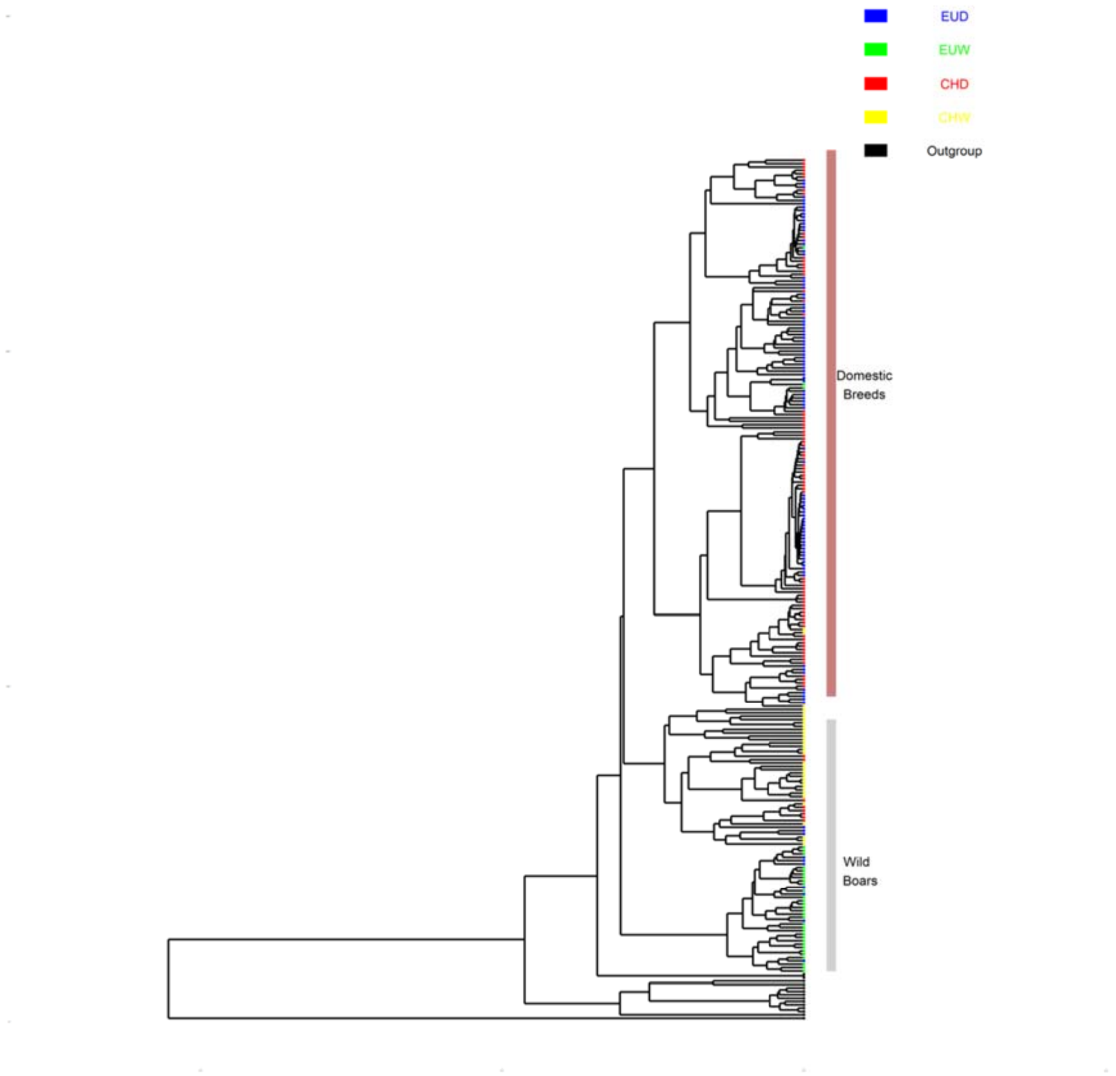

Supplementary Figure 5. Neighbor-joining tree in unidirectional selection regions. Neighbor-joining tree is based on an identity-by-state (IBS) distance matrix, using the data in unidirectional selection regions only. The CHD include Mini-pigs.

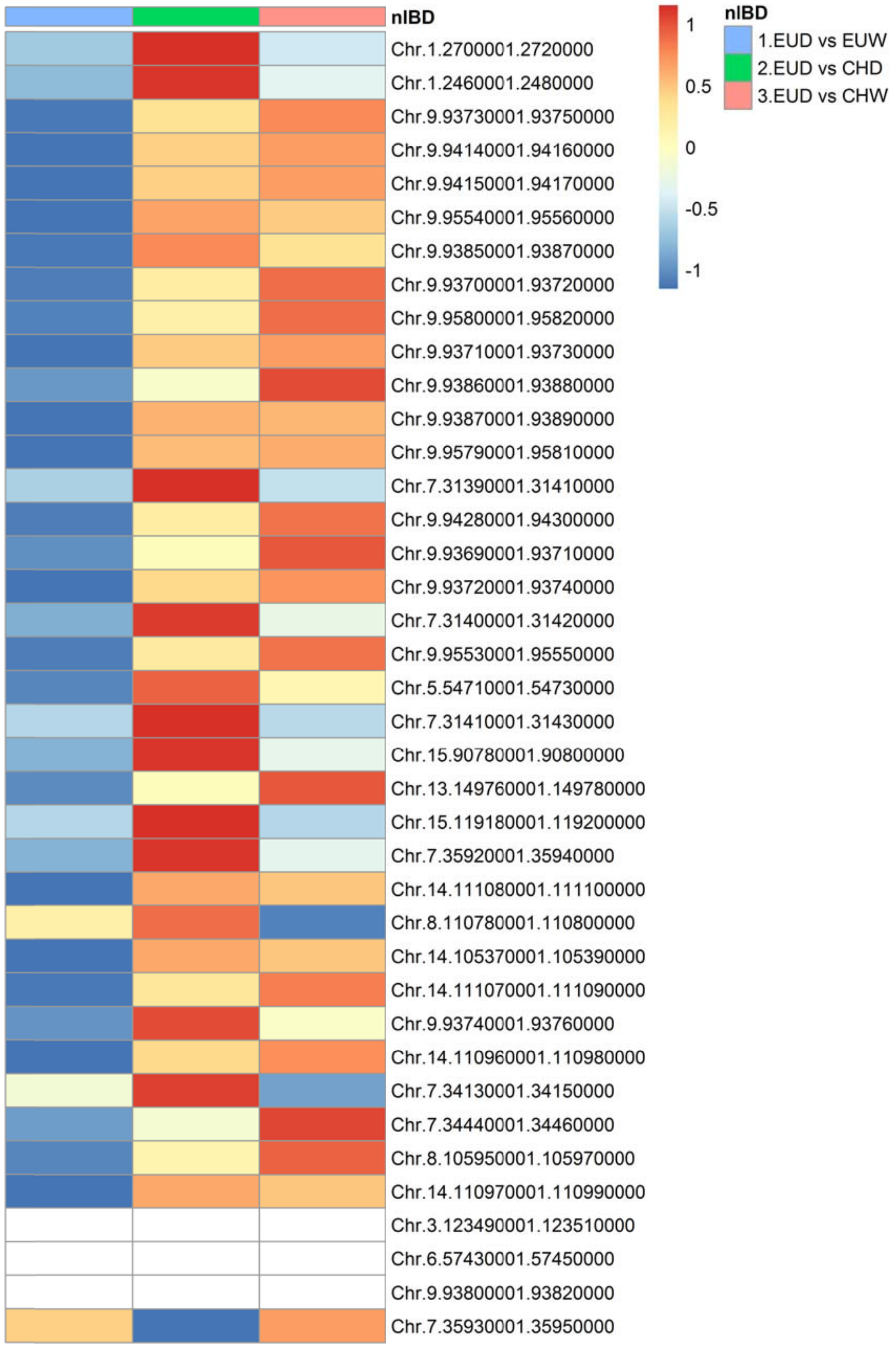

Supplementary Figure 6. Pairwise nIBD between EUD and the other three groups in unidirectional selection regions. Heatmap from pairwise nIBD in the 39 unidirectional regions. The heatmap was plotted scaled by row.

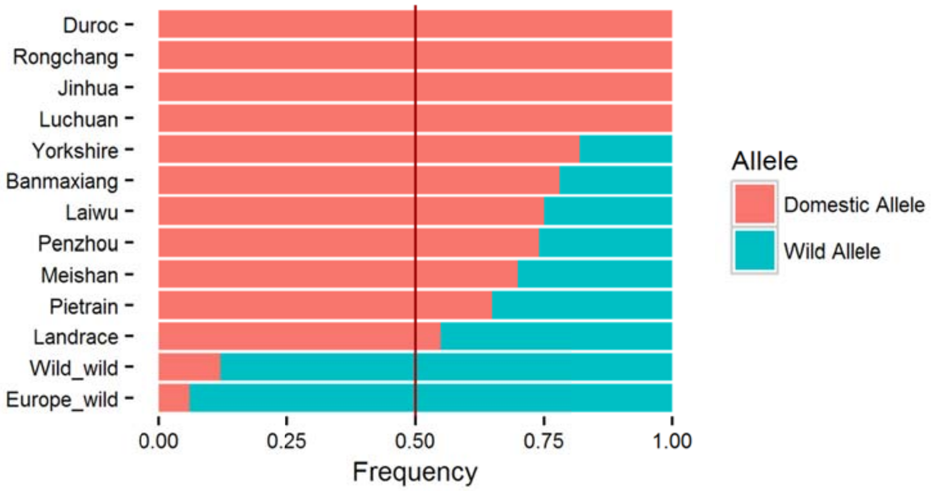

Supplementary Figure 7. The average frequency of the 5 non-synonymous substitutions in each breed. The five variants correspond to mutations of amino acids (P419T, K616Q, G717C, F803L, and T839P) in *AHR*.

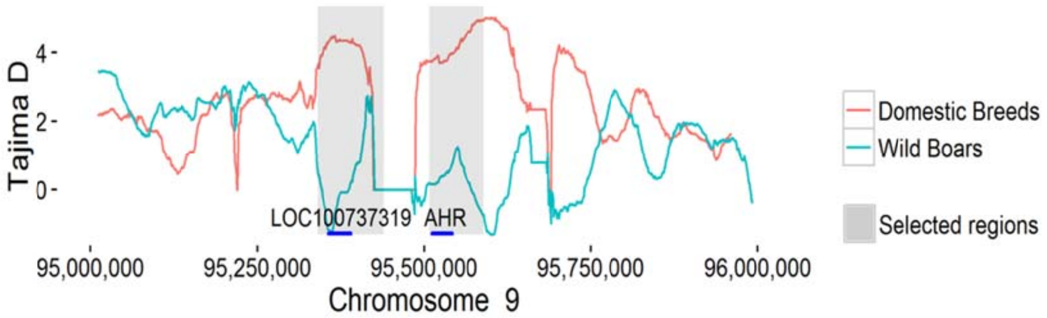

Supplementary Figure 8. Genomic regions with strong unidirectional selection signals in European domestic breeds and European wild boars.

Tajima's D (red and blue lines for domestic breeds and wild boars, respectively) are plotted using a 20 kb sliding window.

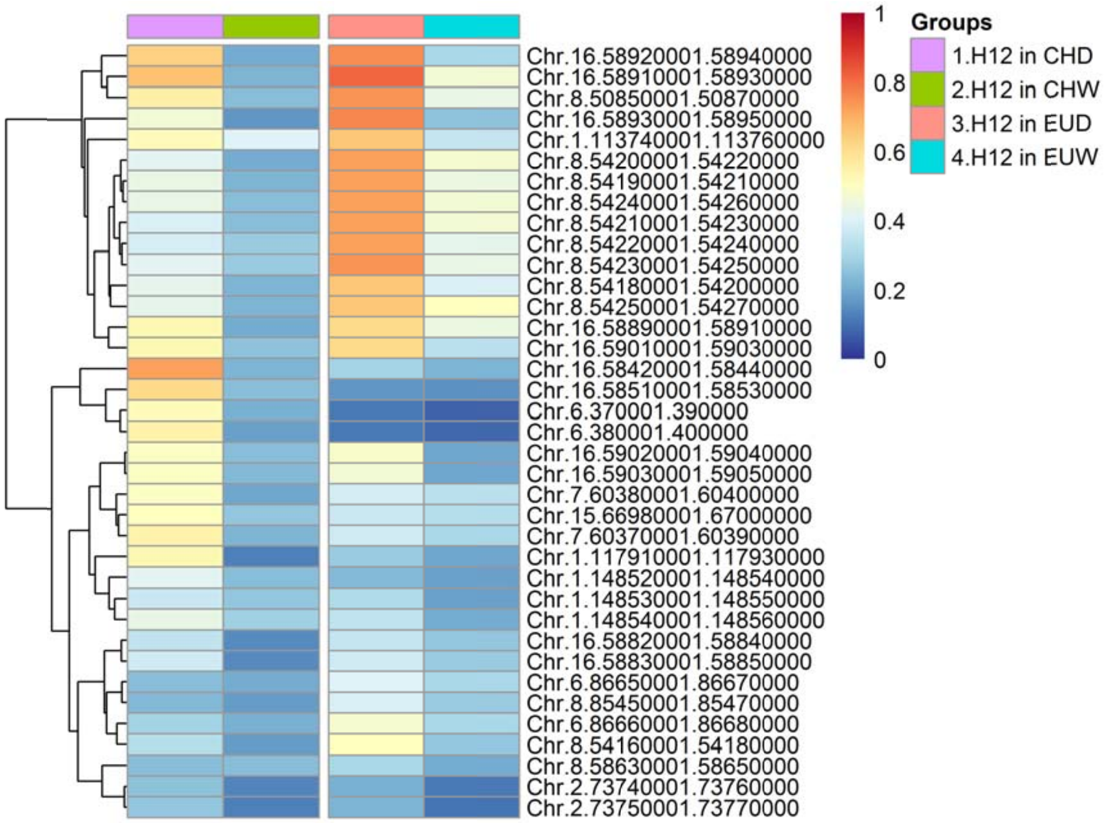

Supplementary Figure 9. The H12 in unidirectional selection regions for each group. Heatmap from hierarchical clustering of the H12 value in the 37 bidirectional regions for each group. Heat-map colors (right) indicate the H12 value.

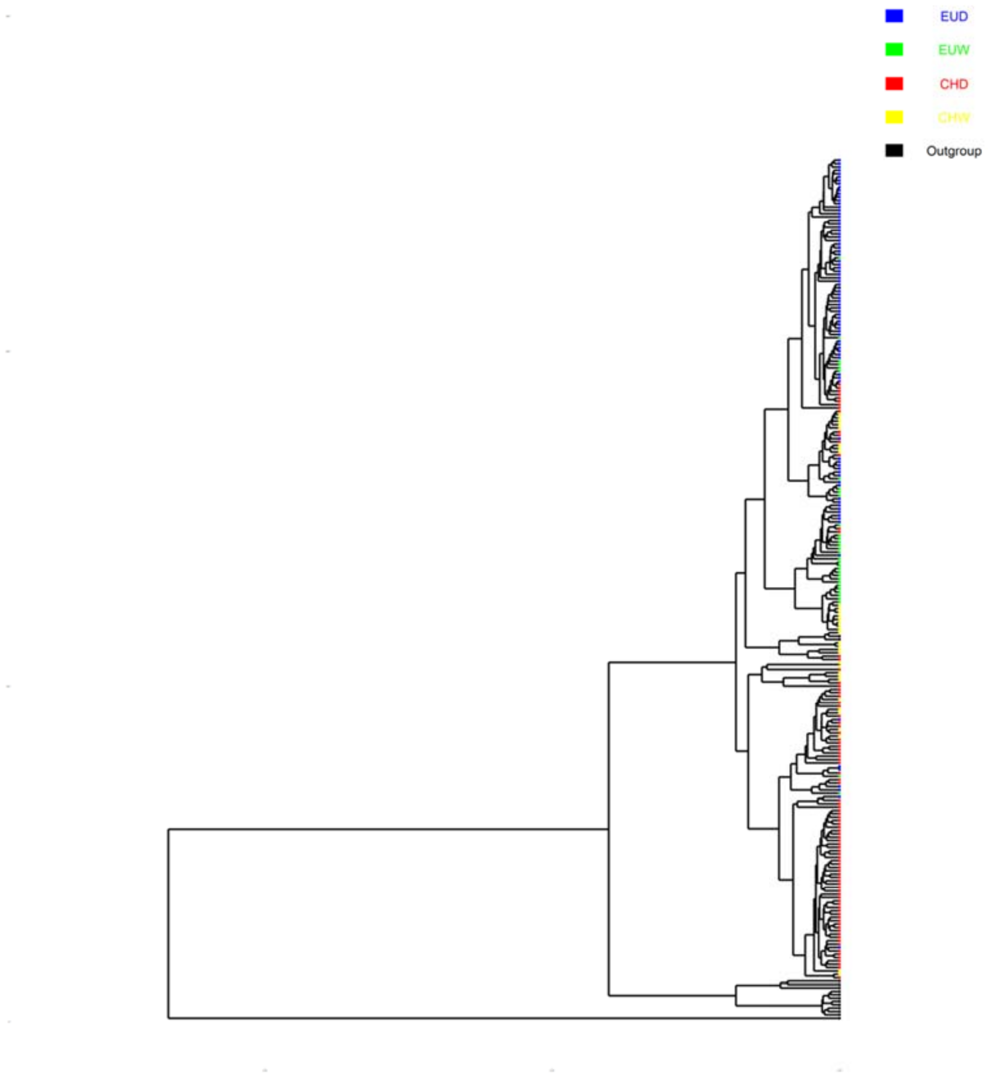

Supplementary Figure 10. Neighbor-joining tree in bidirectional selection regions. Neighbor-joining tree is based on an identity-by-state (IBS) distance matrix, using the data in unidirectional selection regions only. The CHD include Mini-pigs.

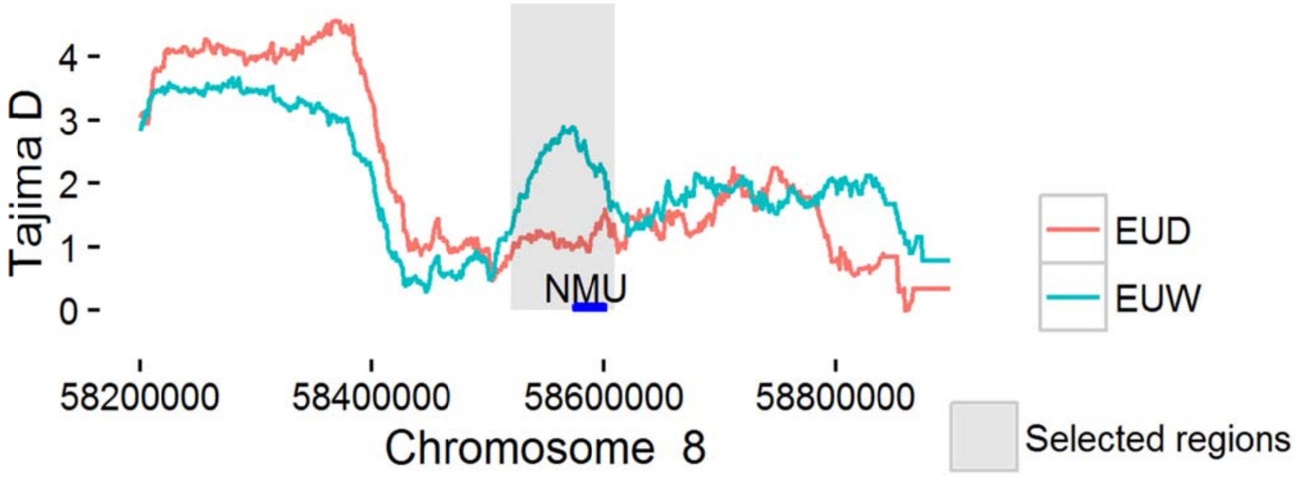

Supplementary Figure 11. Genomic regions with strong bidirectional selection signals in European domestic breeds and European wild boars. Tajima's D (red and blue lines for European domestic breeds and European wild boars, respectively) are plotted using a 20 kb sliding window.

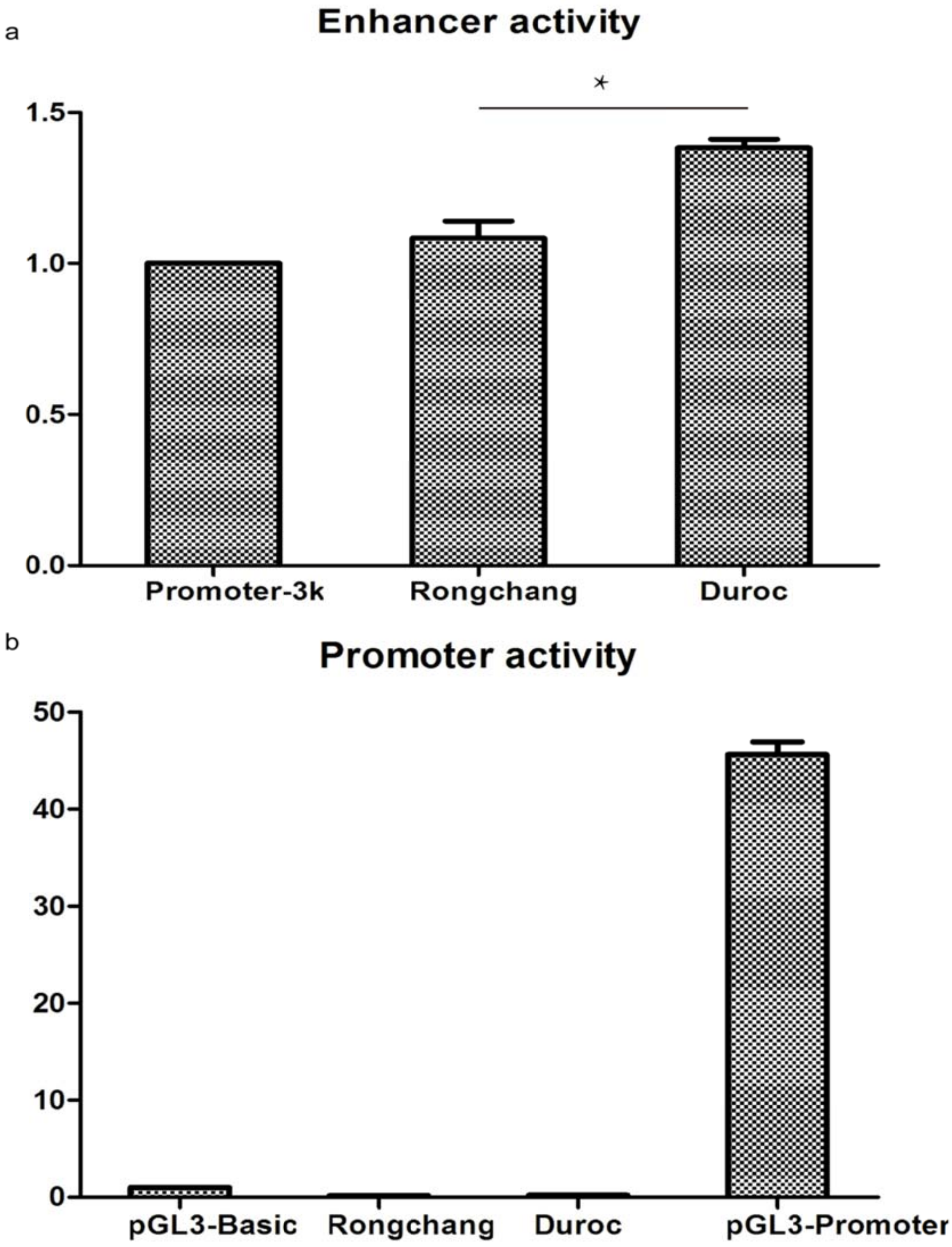

Supplementary Figure 12. Validation the effect of the mutations in *NMU* using pig fibroblast cells. (a), Luciferase assay testing the enhancer activity for different allele in the intron of the *NMU* in pig fibroblast cells**. (b),** Luciferase assay testing the promoter activity for different allele in the intron of the *NMU* in pig fibroblast cells.
